# Mutant NPM1-regulated lncRNA HOTAIRM1 promotes leukemia cell autophagy and proliferation by targeting EGR1 and ULK3

**DOI:** 10.1186/s13046-021-02122-2

**Published:** 2021-10-06

**Authors:** Yipei Jing, Xueke Jiang, Li Lei, Meixi Peng, Jun Ren, Qiaoling Xiao, Yao Tao, Yonghong Tao, Junpeng Huang, Lu Wang, Yuting Tang, Zailin Yang, Zesong Yang, Ling Zhang

**Affiliations:** 1 Key Laboratory of Laboratory Medical Diagnostics Designated by the Ministry of Education, School of Laboratory Medicine, Chongqing Medical University, No.1, Yixueyuan Road, Chongqing, 400016 China; 2 Department of Clinical Laboratory, The Third Affiliated Hospital of Chongqing Medical University, Chongqing, China; 3 Department of Hematology, The First Affiliated Hospital of Chongqing Medical University, Chongqing, China

**Keywords:** Acute myeloid leukemia, Nucleophosmin, Long noncoding RNA, HOTAIRM1, Autophagy, Proliferation, EGR1, ULK3

## Abstract

**Background:**

Acute myeloid leukemia (AML) with mutated nucleophosmin (NPM1), which displays a distinct long noncoding RNA (lncRNA) expression profile, has been defined as a unique subgroup in the new classification of myeloid neoplasms. However, the biological roles of key lncRNAs in the development of NPM1-mutated AML are currently unclear. Here, we aimed to investigate the functional and mechanistic roles of the lncRNA HOTAIRM1 in NPM1-mutated AML.

**Methods:**

The expression of HOTAIRM1 was analyzed with a public database and further determined by qRT-PCR in NPM1-mutated AML samples and cell lines. The cause of upregulated HOTAIRM1 expression was investigated by luciferase reporter, chromatin immunoprecipitation and ubiquitination assays. The functional role of HOTAIRM1 in autophagy and proliferation was evaluated using western blot analysis, immunofluorescence staining, a Cell Counting Kit-8 (CCK-8) assay, a 5-ethynyl-2′-deoxyuridine (EdU) incorporation assay, flow cytometric analyses and animal studies. The action mechanism of HOTAIRM1 was explored through RNA fluorescence in situ hybridization, RNA pulldown and RNA immunoprecipitation assays.

**Results:**

HOTAIRM1 was highly expressed in NPM1-mutated AML. High HOTAIRM1 expression was induced in part by mutant NPM1 via KLF5-dependent transcriptional regulation. Importantly, HOTAIRM1 promoted autophagy and proliferation both in vitro and in vivo. Mechanistic investigations demonstrated that nuclear HOTAIRM1 promoted EGR1 degradation by serving as a scaffold to facilitate MDM2-EGR1 complex formation, while cytoplasmic HOTAIRM1 acted as a sponge for miR-152-3p to increase ULK3 expression.

**Conclusions:**

Taken together, our findings identify two oncogenic regulatory axes in NPM1-mutated AML centered on HOTAIRM1: one involving EGR1 and MDM2 in the nucleus and the other involving the miR-152-3p/ULK3 axis in the cytoplasm. Our study indicates that HOTAIRM1 may be a promising therapeutic target for this distinct leukemia subtype.

**Supplementary Information:**

The online version contains supplementary material available at 10.1186/s13046-021-02122-2.

## Background

Acute myeloid leukemia (AML) is a heterogeneous disease characterized by genetic abnormalities and epigenetic changes [1]. In the 2016 updated World Health Organization (WHO) classification of myeloid neoplasms, AML with nucleophosmin (NPM1) mutations was defined as a distinct subtype [2]. NPM1 mutation results in cytoplasmic mislocalization of the mutant protein (NPM1c +), which is critical for its role in leukemogenesis [3]. To date, researchers have focused mainly on the effect of NPM1c + on functional interactions with its nuclear protein interacting partners and have tried to target NPM1c + by using CRM1 inhibitors or a combination of all-trans retinoic acid (ATRA) and arsenic trioxide (ATO) [4, 5]. Although most patients respond to the current therapy and achieve complete remission, relapse occurs frequently [6]. Hence, novel therapeutic targets should be investigated to improve the diagnosis and treatment of patients with NPM1-mutated AML.

Recently, with the development of high-throughput transcriptome analysis via next-generation sequencing, an increasing number of long noncoding RNAs (lncRNAs) have been discovered [7–9]. LncRNAs comprise a heterogeneous family of RNA molecules longer than 200 nucleotides with no or limited protein-coding potential [10, 11]. Originally considered mere transcriptional noise, lncRNAs are starting to be considered pivotal regulators in numerous biological processes, including cell proliferation, apoptosis, migration, autophagy, and other processes [12–14]. In parallel, lncRNAs are dynamically regulated by intracellular factors, such as transcription factors, as well as by extracellular factors [15]. Accumulating evidence indicates that abnormal expression of specific lncRNAs plays an important role in leukemogenesis [16], and a recent report provided evidence that NPM1-mutated AML displays a distinct lncRNA expression profile [17]. The lncRNA HOX antisense intergenic RNA myeloid 1 (HOTAIRM1), located on chromosome 7p15 between the human HOXA1 and HOXA2 genes, was originally considered a lncRNA associated with differentiation and expressed in myeloid lineage cells [18]. However, recently, HOTAIRM1 has been found to be expressed in multiple other cell types, such as neurons [19] and glioblastoma cells [20]. Dias Beya et al. [21] were the first to reveal in 2015 that HOTAIRM1 is upregulated in patients with NPM1-mutated AML compared to patients with NPM1 non-mutated patients and that expression of HOTAIRM1 is associated with overall survival. Therefore, a deeper exploration of the biological significance of HOTAIRM1 in leukemia is imperative.

In the present study, we first determined that HOTAIRM1 is highly expressed in NPM1-mutated AML. Moreover, HOTAIRM1 expression was at least partially upregulated by mutant NPM1 via Kruppel-like factor 5 (KLF5)-dependent transcriptional regulation. Importantly, HOTAIRM1 promoted leukemia cell autophagy and proliferation in vitro and in vivo. Furthermore, HOTAIRM1 exerted its oncogenic effects by acting as a scaffold to recruit MDM2 to EGR1 in the nucleus and serving as a miRNA sponge to regulate ULK3 in the cytoplasm. Collectively, our findings reveal the oncogenic role of HOTAIRM1 and provide novel insights for future treatment of this distinct leukemia subtype.

## Methods

### Gene expression omnibus (GEO) and the cancer genome atlas (TCGA) analysis

Gene profiling data of AML patients were downloaded from the NCBI Gene Expression Omnibus (GEO) (https://www.ncbi.nlm.nih.gov/gds) under the accession number GSE15434 (*n* = 251) and The Cancer Genome Atlas (TCGA) (http://www.cancergenome.nih.gov) (*n* = 151). To filter out lncRNAs from gene expression matrix, we used R software (version 3.6.3) and R package biomaRt to annotate the obtained data. Differentially expressed lncRNAs were then identified using the limma package. The R packages ggplot2 and pheatmap were used to generate lncRNA expression heatmaps and volcano plots, respectively. Moreover, the overall survival (OS) of AML and NPM1-mutated AML samples from the data of TCGA and BeatAML databases were analyzed according to Kaplan-Meier method.

### Clinical samples

A total of peripheral blood and bone marrow samples of 34 AML patients, including 20 NPM1-unmutated AML and 14 NPM1-mutated AML cases, were obtained from the First Affiliated Hospital of Chongqing Medical University and the Third Affiliated Hospital of Chongqing Medical University. Details of the clinical characteristics of patients are provided in Additional file 1: Table S1. Approval was obtained from the ethics committee of Chongqing Medical University. This study was performed in compliance with the Declaration of Helsinki. Written informed consents were signed by all subjects for study purpose. The mononuclear cells were enriched by Ficoll lymphocyte separation solution (Hao Yang Biological Manufacture Co., Ltd., Tianjin, China). Briefly, the sample was diluted with an equal volume of PBS and mixed well. The blood suspensions were added into the surface of Ficoll and then centrifuged to form discrete layer. The mononuclear layers were collected and washed twice with 1 × PBS. Finally, total RNA from the mononuclear cells was isolated for the analysis of HOTAIRM1 expression.

### Cell culture

Human myeloid leukemia cell lines OCI-AML2 and OCI-AML3 were obtained from Deutsche Sammlung von Mikroorganismen und Zellkulturen GmbH (DSMZ, Braunschweig, NI, Germany). The OCI-AML3 cells carry NPM1 mutation type A (NPM1-mA). Stable NPM1-mA-GFP expressing OCI-AML2 cells (OCI-AML2 + NPM1-mA) were generated (Additional file 6: Figure S1a-c) using lentiviral transduction and selected with 5–10 μg/mL blasticidin and fluorescence-activated cell sorter using a FACSCalibur-Sort instrument (Becton Dickinson Biosciences, Copenhagen, Denmark) equipped with a 15-mW argon ion laser (488 nm) for excitation. Human myeloid leukemia cell lines KG-1a, NB4, THP-1, HL-60, U937 and human embryonic kidney cell line HEK293T were obtained from the American Type Culture Collection (ATCC, Manassas, VA, USA). These myeloid leukemia cell lines were cultured in RPMI-1640 medium (Thermo Fisher Scientific, Waltham, MA, USA) supplemented with 10% fetal bovine serum (FBS, Thermo Fisher Scientific) and 1% penicillin/streptomycin solution (Beyotime, Shanghai, China). HEK293T cells were maintained in DMEM supplemented with 10% FBS and 1% penicillin/streptomycin solution. All cell lines were incubated at 37 °C in the presence of 5% CO_2_.

### Quantitative real-time PCR (qRT-PCR)

Total RNA was isolated using the TRIzol reagent (Takara, Kyoto, Japan), and transcribed into cDNA using the Prime-Script™ RT Reagent Kit (Takara). The qRT-PCR analysis was performed on a CFX Connect™ real-time system (Bio-Rad, Hercules, CA, USA) with the SYBR Green reaction kit (KAPA Biosystems, MA, USA). Cycling conditions were 30 s at 95 °C for the initial denaturation, and amplification was performed with 39 cycles of 5 s at 95 °C, 30 s at 59 °C, 20 s at 72 °C, and finally 10 min at 72 °C for extension. The expression levels were analyzed using the 2^-ΔΔCt^ method. The lncRNA and mRNA expression levels were normalized by GAPDH; the miRNA expression levels were normalized by U6. Details of the primer sequences used are shown in Additional file 2: Table S2. Nuclear export inhibitor KPT-330 (Selleck, Houston, TX, USA) was used to treat OCI-AML3 cells at the concentration of 2 μM for 10 h. These treated cells were applied to detect HOTAIRM1 expression by qRT-PCR.

### Lentiviral vectors and cell infection

The target sequences of the lentivirus-based short hairpin RNA (shRNA) were as follows: shNPM1#1: 5′-GCCGACAAAGATTATCACTTT-3′; shNPM1#2: 5′-AGCAAGGTTCCACAGAAAA-3′; shHOTAIRM1#1: 5′-CTGGAGACTGGTAGCTTATTA-3′; shHOTAIRM1#2: 5′- AGCTGGGAGATTAATCAACCA-3′; shMDM2: 5′- TTGGTATTGCACATTTGCCTG-3′. These shRNA vectors and scramble lentiviral vectors were purchased from Gene Pharma (Shanghai, China). The leukemia cells were infected with lentivirus for 48 h in the presence of 5 μg/mL polybrene (Sigma-Aldrich), after which they were subjected to 2 μg/mL puromycin (Sigma-Aldrich) selection for 7 d. The puromycin-resistant cells were isolated and propagated for further analysis.

### Cell transfection

Plasmids encoding Flag-NPM1-mA was kindly provided by Dr. C.J. Sherr (Genetics and Tumor Cell Biology, St, Jude Children’s Research Hospital, Memphis, TN, USA). The pcDNA3.1 vector expressing HOTAIRM1, EGR1, ULK3, and empty vector were designed and constructed by Genecreate (Wuhan, China). The HOTAIRM1 sequences containing wild-type or mutant EGR1 binding sites were respectively cloned into the pcDNA3.1 vector to form the HOTAIRM1-wild-type vector (HOTAIRM1-WT) and the HOTAIRM1-mutated type vector (HOTAIRM1-Mut1 and HOTAIRM1-Mut2). These expression vectors were also purchased from Genecreate (Wuhan, China). The pCMV3-Flag-MDM2 plasmids were purchased from Sino Biological (Beijing, China). The short interfering RNA (siRNA) targeting EGR1, ULK3 and control siRNA were synthesized by Gene Pharma (Shanghai, China). The sequences of siRNA were as follows: siWWP1: 5′-GAGUUGAUGAUCGUAGAAG-3′; siEGR1: 5′-GCUGCUUCAUCGUCUUCCUCU-3′; siULK3: 5′-GCAGACUUUGGUUUCGCAC-3′; siNC: 5′-UUCUUCGAACGUGUCACGU-3′. Additionally, miR-152-3p mimics (5′-UCAGUGCAUGACAGAACUUGG-3′), mimics NC (5′- UUGUACUACACAAAAGUACUG-3′), miR-152-3p inhibitor (5′-CCAAGUUCUGUCAUGCACUGA-3′), inhibitor NC (5′-CAGUACUUUUGUGUAGUACAA-3′) were synthesized by Gene Pharma. Cells (1 × 10^6^ per well) were plated into a six-well plate and transfected with plasmids or siRNA using Lipofectamine™ 2000 (Invitrogen, Carlsbad, CA, USA) following the manufacturer’s protocol. The transfected cells were harvested (transfection efficiency > 70% ~ 80%) after 48 h to 72 h. These cells were then subjected to further functional assays.

### Western blot analysis

The cultured cells were washed and lysed in cell lysis buffer (Beyotime) with protease inhibitor (Bimake, Houston, TX, USA). Then, total protein concentrations in different samples were measured by a BCA protein assay kit (Beyotime). Subsequently, equal amounts of protein were loaded on a 12% sodium dodecyl sulfate polyacrylamide gel electrophoresis (SDS-PAGE). After the proteins were separated, they were transferred onto polyvinylidene fluoride (PVDF) membranes and subsequently blocked in 5% non-fat milk (Boster Biological Technology, Wuhan, China). Thereafter, the PVDF membranes were immunoblotted with the indicated primary antibodies overnight at 4 °C. The following primary antibodies and respective dilutions were used in this study: anti-NPM1-mA (#PA1–46356, 1:1000; Thermo Fisher Scientific); anti-NPM1-wt (#32–5200, 1:1000; Thermo Fisher Scientific); anti-GAPDH (sc-47,724, 1:5000; Santa Cruz Biotechnology, Dallas, TX, USA); anti-β-tubulin (ab18207, 1:1000; Abcam, Cambridge, United Kingdom), anti-PCNA (ab29, 1:1000; Abcam), anti-KLF5 (ab137676, 1:1000; Abcam), anti-WWP1 (ab43791, 1:1000; Abcam), anti-Cyclin D1 (ab185241, 1:1000; Abcam), anti-Ago2 (ab186733, 1:1000; Abcam), anti-MDM2 (ab16895, 1:1000; Abcam); anti-LC3 (A5179, 1:1000), anti-p62 (A5180, 1:1000), anti-CDK4 (A5189, 1:1000), anti-Bax (A5131, 1:1000), anti-Bcl-2 (A5010, 1:1000) and anti-ULK3 (A5208, 1:1000) were purchased from Bimake; anti-EGR1 (#4154, 1:1000) and anti-ubiquitin (#3936, 1:1000) were purchased from Cell Signaling Technology (Danvers, MA, USA). Then the PVDF membranes were incubated with anti-rabbit secondary antibody (#SA00001–2, 1:4000; Proteintech, Rosemont, IL, USA) or anti-mouse secondary antibody (#A9917, 1:4000; Millipore, Burlington, MA, USA) for 1 h, and subsequently exposed to enhanced chemiluminescence substrate (Millipore). GAPDH was used as the internal control. Protein synthesis inhibitor CHX (Millipore) was incubated with cells for the indicated periods of time. Proteasome inhibitor MG132 (Selleck) was used to treat cells at the concentration of 5 μM for 8 h. These treated cells were applied to detect EGR1 and KLF5 expression by western blotting.

### Luciferase reporter assay

For the HOTAIRM1 promoter luciferase reporter assay, the predicted binding sites of HOTAIRM1 and KLF5 were obtained from JASPAR (http://jaspar.genereg.net/). The different KLF5-binding fragment sequences or the corresponding promoter constructs in the promoter region of HOTAIRM1 were synthesized and then inserted into the pGL3-basic firefly luciferase reporter vector (E1751, Promega), named HOTAIRM1-pGL3-F, HOTAIRM1-pGL3-S1, HOTAIRM1-pGL3-S2. These vectors were co-transfected into AML cells with KLF5 construct or empty vector using transfection reagent (Invitrogen) following the manufacturer’s procedures. For miR-152-3p target gene luciferase reporter assay, the predicted binding sites of HOTAIRM1 and miR-152-3p, miR-152-3p and ULK3 were obtained from starBase (http://starbase.sysu.edu.cn/). First, the HOTAIRM1 sequences containing wild-type or mutant miR-152-3p binding sites were respectively cloned into pmirGLO dual-luciferase vectors to form the HOTAIRM1-wild-type vector (HOTAIRM1-WT) and the HOTAIRM1-mutated type vector (HOTAIRM1-Mut). Similarly, the vectors of ULK3-WT and ULK3-Mut were constructed according to the above method. Next, these vectors were co-transfected into HEK293T cells with miR-152-3p mimics or mimics NC using Lipofectamine 2000 (Invitrogen) following the manufacturer’s procedures. Measurement of luciferase activity was conducted with a Dual-Luciferase Report Assay System (Promega, Madison, WI, USA) at 48 h post-transfection. The relative luciferase activity was calculated by dividing firefly luciferase activity by renilla luciferase activity. All the expression vectors mentioned above were purchased from Genecreate (Wuhan, China).

### Chromatin immunoprecipitation (ChIP) assays

ChIP assays were performed to verify the interaction between KLF5 and the promoter region of HOTAIRM1. EZ-Magna ChIP kit (Millipore) was utilized to carry out this assay. In brief, cells were treated with 1% formaldehyde at 37 °C. After cells were cross-linked for 10 min, 125 nM glycine was added for the termination of crosslinking for 5 min. Thereafter, cells were harvested and sonicated to generate DNA fragments in length of 200 to 1000 bp. Subsequently, the cell lysis was immunoprecipitated overnight at 4 °C with KLF5 antibody (ab137676; Abcam) or the negative control IgG antibody (#12–371; Millipore), respectively. The ChIP primer sequences are listed in Additional file 3: Table S3. These primers were then used for qRT-PCR according to the manufacturer’s instructions. Using the formula 2^[InputCt − TargetCt]^ × 0.1 × 100, the ChIP data were calculated as percentages with respect to the input DNA. The RCR products were loaded into electrophoresis on a 2% agarose gel and observed using ultraviolet light.

### Immunofluorescence staining

Leukemia cells were washed with PBS and cytospun onto coverslips, then fixed with 4% paraformaldehyde for 20 min under room temperature, permeabilized with 0.3% Triton X-100 for 15 min and then blocked for 30 min with 5% of goat serum. The cells were rinsed with PBS and incubated overnight at 4 °C in dilution buffer containing primary antibody rabbit monoclonal antibody LC3 (NB100-2220, 1:200; Novus, Littleton, CO, USA). The cells were washed 3 times with PBS. Then, the cells were incubated with Alexa Fluor fragment of goat anti-rabbit IgG (Beyotime) for 1 h, and 4′,6-diamino-2-phenylindole (DAPI, Beyotime) was used for the nucleus counterstaining. Finally, the cells were visualized using a fluorescence microscope (Nikon, Tokyo, Japan) at 400× magnification.

### Cell proliferation assays

Cell viability was measured with a Cell Counting Kit-8 (CCK-8) assay (Dojindo Laboratories, Kumamoto, Japan). Cells were seeded into a 96-well plate (Corning, NY, USA) at a density of 3 × 10^3^ cells per well with RPMI-1640 containing 10% FBS. Subsequently, 10 μl CCK8 reagent was added into each well and incubated with cells for 2 h. Finally, the absorbance was measured at 450 nm using the microplate reader (BioTeck, CA, USA). When performing a 5-ethynyl-2′-deoxyuridine (EdU) incorporation assay (Ribobio, Guangzhou, China), we seeded cells into a 24-well plate at a density of 2 × 10^4^ cells per well and each well was added with 50 μM EdU at 37 °C for 4 h, followed by fixation in 4% formaldehyde at room temperature for 15 min. Next, the cells were treated with 0.2% Triton X-100 at room temperature for 5 min and 100 μL Apollo® mixture (RiboBio) for 30 min, and then DAPI was added for 30 min in order to label the cell nuclei. The ratio of EdU-stained cells (with green fluorescence) to DAPI-stained cells (with blue fluorescence) was used to evaluate cell proliferation. Images were taken and analyzed with a fluorescence microscope at 100× magnification.

### Flow cytometric analyses

Cell cycle and apoptosis were analyzed by flow cytometry. Propidium (PI) staining was adopted to assess the cell cycle of leukemia cells. Briefly, cells were washed 3 times with PBS, centrifuged, and the supernatant was discarded. The cell concentration was adjusted to approximately 1 × 10^6^ cells/mL after cells were resuspended in PBS, and added with 1 mL of precooled 75% ethanol (− 20 °C) to fix cells at 4 °C for 2 h, followed by centrifugation. The ice ethanol and the supernatant were discarded, and added with 100 μL of RNase A in the dark, water bathed for at 37 °C for 30 min, and then added with 400 μL of PI (Millipore). The cells were then incubated in dark conditions at 4 °C for 30 min, and the cell cycle was determined using flow cytometry at 488 nm. The Annexin V FITC-PI staining assay was used to detect apoptosis. Cytarabine (Ara-C, Sigma) was used to treat OCI-AML3 cells at the concentration of 10 μM for 48 h, OCI-AML2 + NPM1-mA cells at the concentration of 200 nM for 48 h [22]. These cells were harvested and washed with PBS. Apoptosis staining was performed using an Annexin V FITC-PI apoptosis detection kit (BD Biosciences, Piscataway, NJ, USA) according to the manufacturer’s instructions. Stained cells were analyzed using FACSCalibur™ Flow cytometry (BD Biosciences) with Cell-Quest software.

### Subcellular fractionation and fluorescence in situ hybridization (FISH)

To observe the sublocation of HOTAIRM1 in leukemia cells, nuclear and cytoplasmic RNA substrate were separated using PARIS Kit (Ambion, Austin, TX). HOTAIRM1 expression was determined by qRT-PCR with U6 as the nuclear control and GAPDH as the cytoplasmic control. For FISH assay, cells were first grown in 24-well plates for 24 h and then fixed with 4% paraformaldehyde for 20 min at room temperature. After permeabilization with paraformaldehyde, cells were hybridized with 2 μM Cy3-labeled HOTAIRM1 FISH probe mix (Gene Pharma), and DAPI was used for nuclear counterstaining. Images were taken and analyzed with a fluorescence microscope at 400× magnification.

### RNA pulldown assays

RNA pulldown assays were performed using the RNA-Protein Pull-Down Kit (Thermo Fisher Scientific) according to the manufacturer’s instructions. The cells were crosslinked in 1% formaldehyde for 10 min, equilibrated in glycine buffer for 5 min, washed with cold PBS 3 times, scraped with 1 mL of lysis buffer and incubated for 10 min. The cell samples were sonicated and then centrifuged, after which the supernatant was transferred to a 2-mL tube, and 50 μL cell lysis was saved for input analysis. The lysate supernatant was incubated with HOTAIRM1 probes or a negative probe for 3 h at room temperature with rotation. Then, 100 μL of Streptavidin Magnetic Beads was added, and the mixture was incubated for 1 h with stirring. The bead/sample mixture was washed twice, after which 10% of the mixture was subjected to RNA purification, while the remaining 90% was subjected to protein purification. After washing for 3–4 times, HOTAIRM1-associated proteins, which were retrieved from beads, were subjected to SDS-PAGE and silver staining. Differential protein bands were excised and identified by mass spectrometry and western blotting.

### RNA immunoprecipitation (RIP) assays

RIP assays were performed using an EZ-Magna RIP™ RNA-Binding Protein Immunoprecipitation Kit (Millipore) according to the manufacturer’s instructions. The cells were rinsed with cold PBS and fixed in 1% formaldehyde for 10 min. After centrifugation (1500×g for 15 min at 4 °C), cell pellets were collected and re-suspended in RIP lysis buffer. Then 100 μL of the lysate was incubated with RIP buffer containing magnetic beads, which were conjugated with human anti-EGR1 (Cell Signaling Technology), anti-Ago2 (Abcam) and normal rabbit IgG (Millipore). Among the antibodies, IgG was considered a negative control (NC). Proteinase K buffer was then added to the samples. Finally, the target RNA was extracted and purified for further study by qRT-PCR.

### Immunoprecipitation (IP) assay

The cells were harvested and washed twice with ice-cold PBS buffer. Cells were then sonicated in IP buffer [20 mM Tris-Cl, 150 mM NaCl, 1 mM EDTA, 1 mM EGTA, 1% (v/v) Triton X-100, 2.5 mM sodium pyrophosphate, 1 mM β-glycerolphosphate, 1 mM Na_3_VO_4_, and protease inhibitor cocktail (Bimake), pH 7.5] at 4 °C for 3 times per 5 s by Bio-ruptor UCD-200 (Diagenode, Belgium), followed by centrifuged at 13,300 g at 4 °C for 10 min to remove the cell debris. Expressing of the indicated proteins in the lysates was checked by western blotting using relevant antibodies to normalize total amounts of the inputs. The supernatants were each incubated with specific antibodies or normal IgG (as control), and equal amounts of protein A/G beads (Bimake) overnight at 4 °C. The protein A/G beads with the bound antibodies and interacting proteins were pelleted and washed 3 times with IP buffer before boiled in 2× SDS-PAGE sample. The boiled samples were then resolved in SDS-PAGE and subject to western blot analysis.

### Animal experiments

Five-week-old female NOD/SCID mice were purchased from The Animal Resources Centre (Canning Vale WA, Australia). NOD/SCID mice were randomly split in 3 groups (*n* = 5/group). Total of 2 × 10^7^ engineered OCI-AML3 cells stably transfected with shNC, shHOTAIRM1 and HOTAIRM1-silenced OCI-AML3 cells followed by HOTAIRM1 plasmid introduction (shHOTAIRM1 + HOTAIRM1) were injected into mice via the tail vein. Mice were monitored weekly for signs of weight loss or lethargy. Peripheral blood was obtained from mice and white blood cell counts (WBC) were analyzed. Mice were killed after 8 weeks, and bone marrow cells were harvested from femurs for wright’s staining and western blotting. Mouse livers and spleens were excised and serially sectioned into 4 μm sections, and then the infiltration of liver and spleen was analyzed by hematoxylin and eosin staining (HE) and Immunohistochemistry (IHC) analysis of Ki-67. The Kaplan-Meier method was used to analyze the survival curves of mice in each group. All experimental procedures involving animals were in accordance with the Guide for the Care and Use of Laboratory Animals (NIH publication no. 80–23, revised 1996) and were performed according to the institutional ethical guidelines for animal experiments.

### Statistical analysis

Data from at least three independent experiments were presented as the mean ± standard deviation (SD). The SPSS (Version 17.0) and GraphPad (Prism 7.0) were used to calculate statistical significance. Differences between multiple groups were evaluated using one-way analysis of variance (ANOVA). Differences between two groups were compared using the unpaired Student’s t test. The Kaplan-Meier estimation and the log-rank test were used to compare the survival difference. *P* < 0.05 was considered statistically significant (*P < 0.05, **P < 0.01, ***P < 0.001).

## Results

### HOTAIRM1 is highly expressed in NPM1-mutated AML cells

**Fig. 1:**
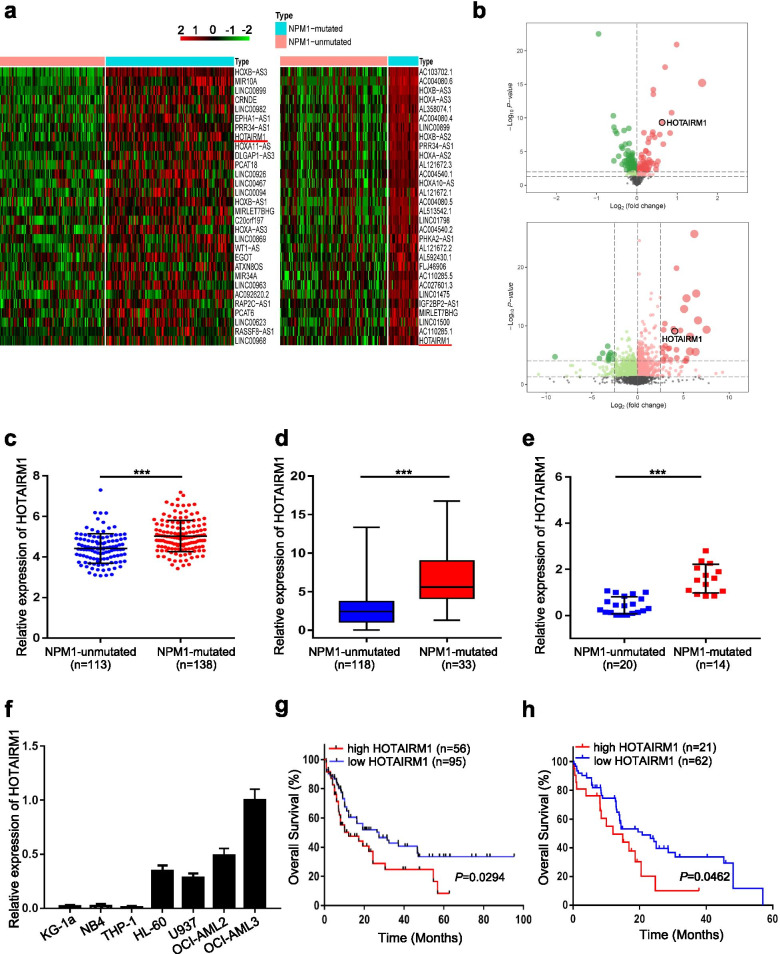
High expression levels of HOTAIRM1 in NPM1-mutated AML cells. **a** Heat maps of the top 30 lncRNAs upregulated in NPM1-mutated AML samples compared to NPM1-wt AML samples (left, GSE15434; right, TCGA). **b** Volcano plots showing the expression profiles of lncRNAs (top, GSE15434; bottom, TCGA). **c** HOTAIRM1 expression levels in patients with NPM1-wt AML (*n* = 113) and patients with NPM1-mutated AML (*n* = 138) were obtained from the GSE15434 dataset. **d** HOTAIRM1 expression levels in AML patients without (*n* = 118) and with NPM1 mutation (*n* = 33) were obtained from the TCGA database. **e** The expression levels of HOTAIRM1 in primary AML blasts without (*n* = 20) and with NPM1 mutation (*n* = 14). **f** qRT-PCR was conducted to determine the level of HOTAIRM1 in AML cell lines. GAPDH was used as the endogenous control. **g-h** Survival analysis based on HOTAIRM1 expression in AML (**g**) and NPM1-mutated AML samples (**h)** using the data from TCGA and BeatAML. The data are presented as the mean ± SD of three independent experiments. ***P < 0.001

To identify the lncRNAs that potentially participate in the progression of NPM1-mutated AML, we analyzed gene expression profiles using public databases (GSE15434 and TCGA datasets). As expected, many lncRNAs were identified to be differentially expressed in NPM1-mutated AML, among which HOTAIRM1 was one of the most substantially changed (Fig. 1a-b). Notably, HOTAIRM1 was significantly upregulated in AML with NPM1 mutation compared to AML without NPM1 mutation (Fig. 1c-d). Consistent with these findings, the expression level of HOTAIRM1 was measured by qRT-PCR in 34 primary AML blasts, and HOTAIRM1 was found to be highly expressed in NPM1-mutated AML cases (*n* = 14) compared with NPM1- unmutated AML cases (*n* = 20) (Fig. 1e). Moreover, the HOTAIRM1 expression level was measured in a panel of AML cell lines, and relatively high expression of HOTAIRM1 was observed in OCI-AML3 cells, which naturally carry NPM1 mutation type A (NPM1-mA) (Fig. 1f). Subsequently, the prognostic value of HOTAIRM1 was assessed using data from TCGA and BeatAML. Kaplan-Meier survival analysis of AML patients revealed a significantly shorter overall survival time in the cohort with high HOTAIRM1 expression (Fig. 1g). Moreover, high HOTAIRM1 expression was evidently associated with poorer prognosis in NPM1-mutated AML (Fig. 1h). These findings demonstrated the high expression of HOTAIRM1 in NPM1-mutated AML.

### High expression of HOTAIRM1 is maintained by mutant NPM1 via KLF5-dependent transcriptional regulation

**Fig. 2:**
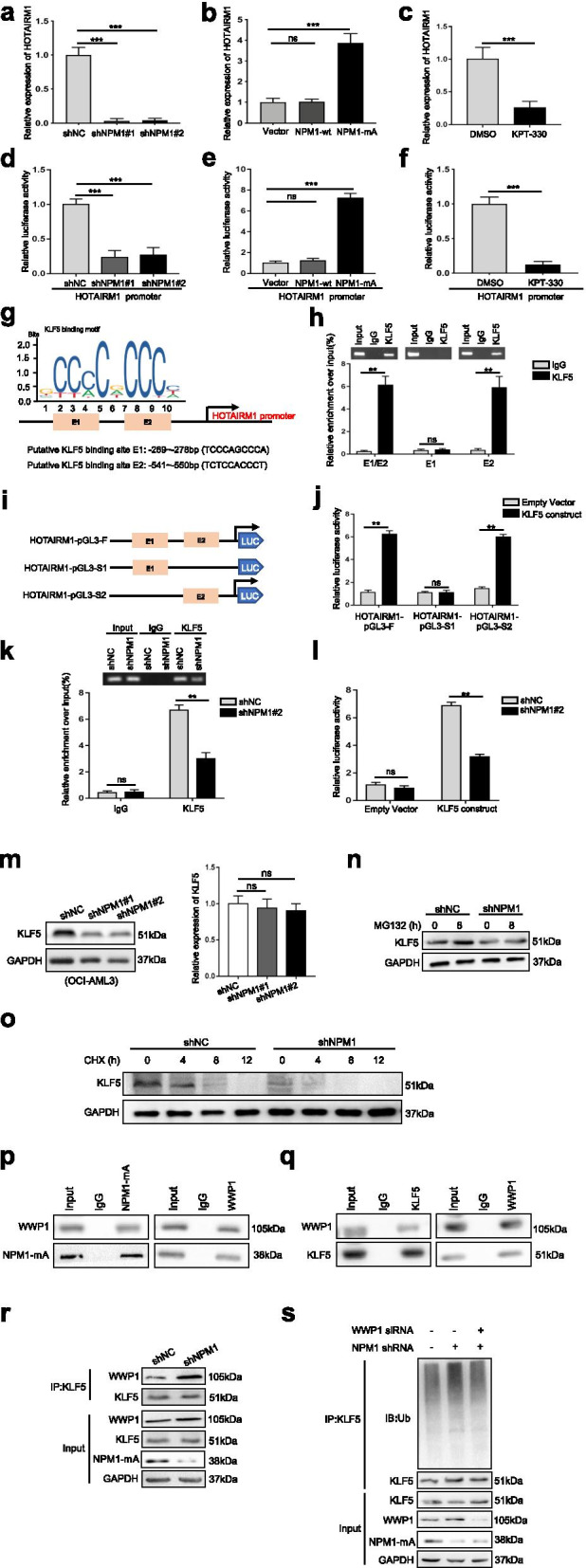
HOTAIRM1 expression is induced by mutant NPM1 via KLF5-dependent transcriptional regulation. **a-b** HOTAIRM1 expression was measured by qRT-PCR in OCI-AML3 cells transfected with shNPM1 (**a**) and in OCI-AML2 cells transfected with the NPM1-mA plasmid (**b**). **c** qRT-PCR was conducted to determine the level of HOTAIRM1 in OCI-AML3 cells treated with KPT-330. **d-e** Relative luciferase activity was analyzed in NPM1-mA-silenced OCI-AML3 cells transfected with the HOTAIRM1 promoter construct (**d**) and NPM1-mA-enforced OCI-AML2 cells transfected with the HOTAIRM1 promoter construct (**e**). **f** Luciferase reporter assays were performed with KPT-330-treated OCI-AML3 cells using the indicated reporters. **g** Prediction of KLF5 binding sites in the HOTAIRM1 promoter region using JASPAR. **h** Enrichment of KLF5 on the E2 fragment of the HOTAIRM1 promoter was measured by ChIP in OCI-AML3 cells. **i** Construction of the luciferase reporter vectors HOTAIRM1-pGL3-F (containing all KLF5 binding sites), HOTAIRM1-pGL3-S1 (containing the sites at bp − 269 and − 278) and HOTAIRM1-pGL3-S2 (containing the sites at bp − 541 and − 550). **j** Luciferase reporter assays of OCI-AML3 cells transfected with the HOTAIRM1-pGL3-F, HOTAIRM1-pGL3-S1, or HOTAIRM1-pGL3-S2 vector, the KLF5 construct, or empty vector. **k** Enrichment of KLF5 on the HOTAIRM1 promoter in OCI-AML3 cells treated with or without shNPM1 was detected by ChIP. **l** HOTAIRM1 promoter activity in OCI-AML3 cells treated with or without shNPM1 was detected by luciferase reporter assays. **m** Western blot (left) and qRT-PCR (right) analyses of KLF5 expression in OCI-AML3 cells transduced with control shRNA or shNPM1. **n** KLF5 protein expression in transfected OCI-AML3 cells after treatment with MG132. **o** KLF5 expression in transfected OCI-AML3 cells treated with CHX. **p-q** IP assays were used to determine the binding of NPM1-mA to WWP1 (**p**) and the binding of KLF5 to WWP1 (**q**) in OCI-AML3 cells. **r** The binding of WWP1 to KLF5 in NPM1-silenced OCI-AML3 cells was investigated by IP assays. **s** Western blot analysis of endogenous KLF5 ubiquitination in transfected OCI-AML3 cells followed by siWWP1 and/or shNPM1. The data are presented as the mean ± SD of three independent experiments. **P < 0.01, ***P < 0.001. n.s. indicates no significant difference

Next, we explored the reason for the high HOTAIRM1 expression in NPM1-mutated AML cells. The NPM1 mutation is an AML driver lesion [23], and mutant NPM1 aberrantly mislocalized in the cytoplasm may induce mislocalization of its interacting partners, such as proteins and RNA molecules [22]. We first observed the subcellular localization of HOTAIRM1 in OCI-AML2 cells, OCI-AML3 cells and engineered OCI-AML2 + NPM1-mA cells. Cell fractionation experiments showed that the cellular localization of HOTAIRM1 remained the same regardless of the NPM1 mutational status (Additional file 7: Figure S2). Subsequently, we investigated the impact of this mutation on the expression of HOTAIRM1. Knockdown of NPM1-mA (Additional file 8: Figure S3a-b) significantly decreased the HOTAIRM1 level (Fig. 2a), whereas overexpression of NPM1-mA (Additional file 8: Figure S3c) had the opposite effect (Fig. 2b). Based on the effects of NPM1 mutation on the aberrant cytoplasmic localization of the NPM1 protein [24], KPT-330, a selective CRM-1/exportin-1 (XPO-1) inhibitor, was used to relocalize cytoplasmic NPM1-mA to the nucleus (Additional file 8: Figure S3d), resulting in a decrease in the level of HOTAIRM1 (Fig. 2c). Furthermore, we sought to determine the molecular mechanism by which NPM1-mA upregulates HOTAIRM1 expression. We first found that either NPM1-mA knockdown or KPT-330 treatment decreased (Fig. 2d, f) but NPM1-mA overexpression increased (Fig. 2e) the luciferase activity in cells transfected with the HOTAIRM1 promoter. NPM1 may bind directly to the promoters of genes (i.e., *c-MYC*) to regulate their expression [25]. However, in our study, neither NPM1-wt nor NPM1-mA was able to bind the promoter of the HOTAIRM1 gene, as determined by ChIP (Additional file 9: Figure S4). We then sought to determine whether the regulation of HOTAIRM1 by NPM1-mA requires a cooperative event involving in this process. KLF5 was predicted to bind to the HOTAIRM1 promoter region with a high score (Additional file 4: Table S4), and the potential KLF5 binding sites in the HOTAIRM1 promoter sequence are shown in Fig. 2g. The ChIP assays showed that the E2 fragment of HOTAIRM1 (from − 541 to − 550 bp) was responsible for the affinity of KLF5 for the HOTAIRM1 promoter (Fig. 2h, Additional file 10: Figure S5). Then, luciferase reporter assays were performed by cotransfecting three different luciferase reporter plasmids (Fig. 2i) with the KLF5 construct. Only the HOTAIRM1-pGL3-S2 group exhibited high HOTAIRM1 promoter activity (Fig. 2j). Notably, knockdown of NPM1-mA led to significant inhibition of the binding between KLF5 and the HOTAIRM1 promoter (Fig. 2k) and thus reduced HOTAIRM1 promoter activity (Fig. 2l), indicating that HOTAIRM1 expression is at least partially upregulated by mutant NPM1 via KLF5-dependent transcriptional regulation.

Finally, we sought to determine the mechanism by which NPM1-mA regulates KLF5 transcriptional activity. The IP-western blotting results showed a lack of binding between NPM1 and KLF5 (Additional file 11: Figure S6a-b). Notably, NPM1-mA knockdown caused a decrease in the KLF5 protein level but not the KLF5 mRNA level (Fig. 2m). In addition, we evaluated KLF5 expression in OCI-AML3 cells incubated with the proteasome inhibitor MG132 (Fig. 2n) and the protein synthesis inhibitor cycloheximide (CHX) (Fig. 2o) after knockdown of NPM1-mA. The results indicated that NPM1-mA regulates KLF5 protein stability via the proteasome pathway. WWP1 is a well-known E3 ubiquitin ligase that targets the KLF5 protein for ubiquitin-mediated degradation [26], and we investigated whether NPM1-mA regulates KLF5 via WWP1. IP experiments showed that NPM1-mA (but not NPM1-wt, Additional file 11: Figure S6c) bound to WWP1 (Fig. 2p) and that WWP1 bound to KLF5 (Fig. 2q). Importantly, NPM1-mA deficiency promoted the protein interaction between WWP1 and KLF5 (Fig. 2r) and increased WWP1-induced KLF5 ubiquitination (Fig. 2s). Collectively, these data showed that high expression of HOTAIRM1 was maintained by mutant NPM1 via KLF5-dependent transcriptional regulation and that WWP1 may be involved in this process.

### HOTAIRM1 promotes leukemia cell autophagy and proliferation

**Fig. 3:**
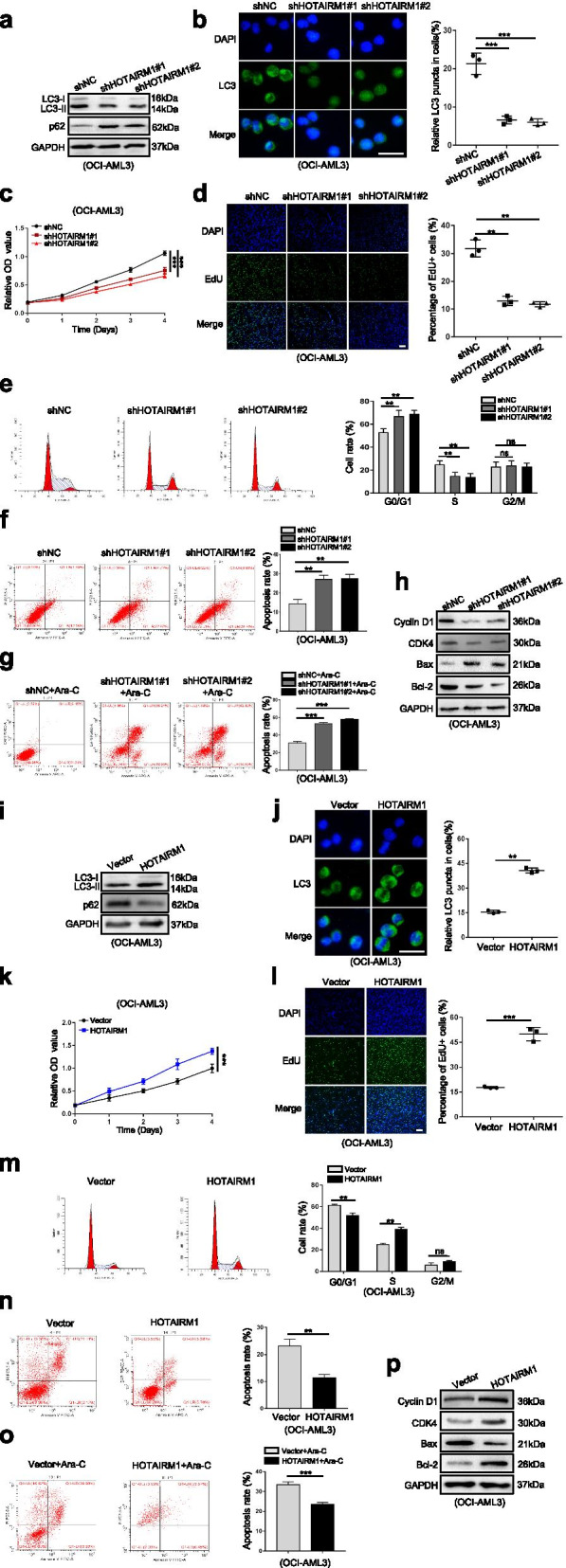
HOTAIRM1 promotes leukemia cell autophagy and proliferation. **a, i** Western blot analysis of LC3-II and p62 levels in OCI-AML3 cells after transfection of shHOTAIRM1 (**a**) and HOTAIRM1 expression vector (**i**). **b, j** Representative microscopy images of LC3 puncta in HOTAIRM1-silenced OCI-AML3 (**b**) and HOTAIRM1-enforced OCI-AML3 (**j**). The bar graphs showed the quantification of the fluorescent puncta data. Scale bar: 25 μm. **c, k** Evaluation of cell viability in OCI-AML3 cells transfected with shHOTAIRM1 (**c**) or HOTAIRM1 plasmid (**k**) for indicated by CCK-8 assays. **d, l** Evaluation of cell proliferation in HOTAIRM1-silenced (**d**) and HOTAIRM1-enforced OCI-AML3 cells (**l**) for indicated by EdU assays. The bar graphs showed the percentage of EdU positive cells. Scale bar: 100 μm. **e, m** Flow cytometry was performed to assess cell cycle of HOTAIRM1-silenced (**e**) and HOTAIRM1-enforced OCI-AML3 cells (**m**). The bar graph shows the percentages of G0/G1-, S-, and G2/M-phase cells. **f, n** Flow cytometry was used to detect the apoptosis of HOTAIRM1-silenced (**f**) and HOTAIRM1- overexpressing OCI-AML3 cells (**n**). LL, dead cells; UL, viable cells; LR, early apoptotic cells; UR, late apoptotic cells. **g, o** Flow cytometry analysis was performed to analyze apoptosis of OCI-AML3 cells after transfection with shHOTAIRM1 (**g**) or HOTAIRM1 plasmid (**o**), followed by treatment with 10 μM Ara-C for 48 h. **h, p** The protein levels of Cyclin D1, CDK4, Bax and Bcl-2 in HOTAIRM1-silenced (**h**) and HOTAIRM1-enforced OCI-AML3 cells (**p**). The data are presented as the mean ± SD of three independent experiments. **P < 0.01, ***P < 0.001. n.s. indicates no significant difference

Then, we investigated the biological functions of HOTAIRM1 in NPM1-mutated AML cells. Two individual shRNAs targeting HOTAIRM1 suppressed HOTAIRM1 expression in OCI-AML3 cells (Additional file 12: Figure S7a) and OCI-AML2 + NPM1-mA cells (Additional file 12: Figure S7c). Next, western blot analysis showed that HOTAIRM1 knockdown significantly decreased the microtubule-associated protein 1 light chain 3 (LC3-II) level and increased the p62 level (Fig. 3a, Additional file 13: Figure S8a). Additionally, immunofluorescence staining indicated that depletion of HOTAIRM1 reduced the accumulation of LC3 puncta (Fig. 3b, Additional file 13: Figure S8b). Then, CCK-8 assays showed that suppression of HOTAIRM1 expression decreased cell viability (Fig. 3c, Additional file 13: Figure S8c). Correspondingly, the number of EdU-positive cells was decreased in the HOTAIRM1-silenced group (Fig. 3d, Additional file 13: Figure S8d). Furthermore, we examined whether HOTAIRM1 affects cell proliferation by altering cell cycle progression and apoptosis. Flow cytometric analyses showed that loss of HOTAIRM1 significantly increased the percentage of cells in G0/G1 phase and reduced that of cells in S phase (Fig. 3e, Additional file 13: Figure S8e). In addition, both the basal (Fig. 3f, Additional file 13: Figure S8f) and Ara-C-induced (Fig. 3g, Additional file 13: Figure S8g) apoptosis rates were markedly increased in HOTAIRM1-silenced cells. Moreover, HOTAIRM1 knockdown significantly decreased the levels of Cyclin D1, CDK4 and Bcl-2 but increased the level of Bax (Fig. 3h, Additional file 13: Figure S8h).

In complementary gain-of-function studies, the HOTAIRM1 expression vector was transfected into OCI-AML3 cells (Additional file 12: Figure S7b) and OCI-AML2 + NPM1-mA cells (Additional file 12: Figure S7d), and these cells were used for further analysis. As anticipated, forced expression of HOTAIRM1 increased the LC3-II level, decreased the p62 level (Fig. 3i, Additional file 13: Figure S8i) and induced the accumulation of LC3 puncta (Fig. 3j, Additional file 13: Figure S8j). Moreover, overexpression of HOTAIRM1 enhanced cell viability and increased the number of EdU-positive cells (Fig. 3k-l, Additional file 13: Figure S8k-l). In addition, gain of HOTAIRM1 promoted cell cycle progression (Fig. 3m, Additional file 13: Figure S8m) and inhibited both basal (Fig. 3n, Additional file 13: Figure S8n) and Ara-C-induced apoptosis (Fig. 3o, Additional file 13: Figure S8o). Furthermore, HOTAIRM1 overexpression significantly increased the levels of Cyclin D1, CDK4 and Bcl-2 but decreased the level of Bax (Fig. 3p, Additional file 13: Figure S8p). Additionally, overexpression of HOTAIRM1 in OCI-AML2 (Additional file 12: Figure S7e) promoted cell autophagy and proliferation (Additional file 14: Figure S9a-g). Collectively, these results indicated an important role of HOTAIRM1 in promoting autophagy and proliferation in NPM1-mutated AML cells.

### Nuclear HOTAIRM1 promotes EGR1 degradation through MDM2-mediated ubiquitination

**Fig. 4:**
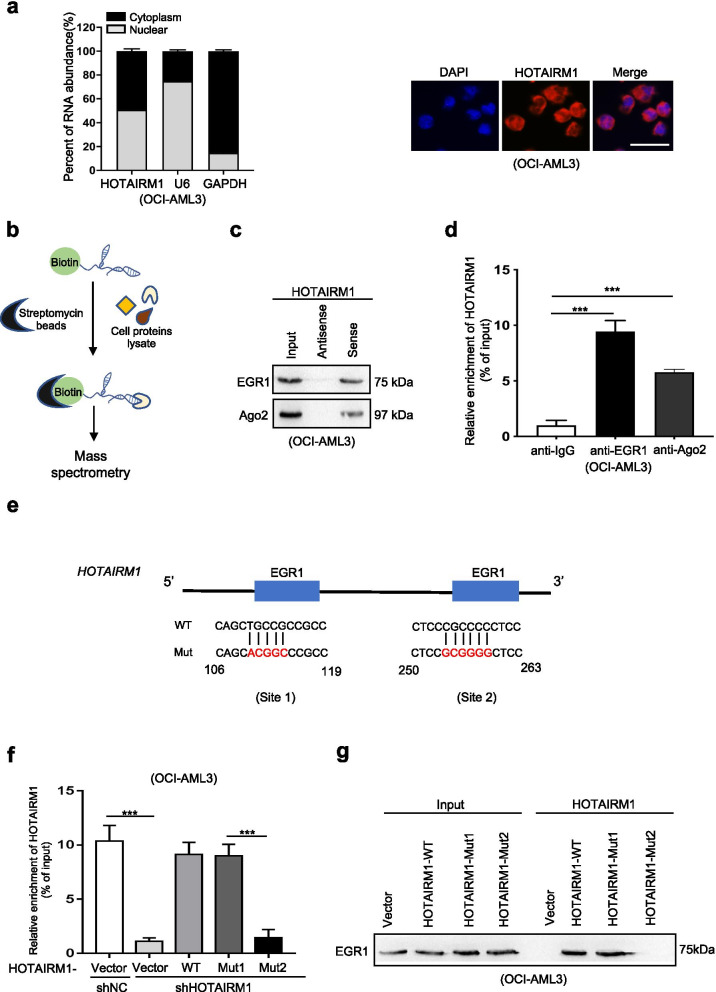
HOTAIRM1 interacts with EGR1 in NPM1-mutated AML cells. **a** The subcellular localization of HOTAIRM1 in OCI-AML3 cells was detected by FISH. Scale bar: 25 μm. **b** Schematic diagram of the identification of HOTAIRM1 binding proteins by RNA pulldown coupled with mass spectrometry. **c** The interactions between EGR1 or Ago2 and HOTAIRM1 in OCI-AML3 cells were validated by RNA pulldown and subsequent western blotting. **d** HOTAIRM1 RNA was measured by qRT-PCR in IgG control, EGR1, and Ago2 RIP samples. **e** Schematic diagram of the putative EGR1-binding sites in HOTAIRM1. **f** The effect of overexpression of HOTAIRM1-WT, HOTAIRM1-Mut1 and HOTAIRM1-Mut2 in HOTAIRM1-silenced OCI-AML3 cells was assessed by RIP assays. **g** RNA pulldown and western blotting were used to detect EGR1 expression in HOTAIRM1-silenced OCI-AML3 cells transfected with different vectors. The data are presented as the mean ± SD of three independent experiments. *P < 0.05, **P < 0.01, ***P < 0.001. n.s. indicates no significant difference

We further explored the potential mechanisms by which HOTAIRM1 contributes to the malignant phenotypes of NPM1-mutated AML cells. First, cellular fractionation and FISH assays were conducted to observe the subcellular localization of HOTAIRM1 in OCI-AML3 cells, and the data showed that HOTAIRM1 was expressed in both the nucleus and cytoplasm (Fig. 4a). Since nuclear lncRNAs can perform their functions by binding to proteins [27, 28], we conducted RNA pulldown assays followed by mass spectrometry in OCI-AML3 cells using an in vitro biotinylated HOTAIRM1 transcript (Fig. 4b). Mass spectrometry analysis (Additional file 5: Table S5) revealed that early growth response 1 (EGR1) bound to HOTAIRM1, and this binding was further confirmed by RNA pulldown followed by western blot analysis (Fig. 4c). EGR1, a zinc finger transcription factor, plays an important role in controlling inflammation, growth, differentiation, apoptosis and tumor progression [29]. Our RIP assays demonstrated that HOTAIRM1 RNA was precipitated with the anti-EGR1 antibody (Fig. 4d). To further identify the EGR1-interacting region in HOTAIRM1, we first generated HOTAIRM1-WT, HOTAIRM1-Mut1 and HOTAIRM1-Mut2 constructs (Additional file 15: Figure S10) based on the JASPAR database (Fig. 4e). Subsequent RIP assays showed that overexpression of HOTAIRM1-Mut1 reversed the inhibition of binding between HOTAIRM1 and EGR1 induced by HOTAIRM1 knockdown (Fig. 4f). Similarly, RNA pulldown assays revealed that EGR1 bound to HOTAIRM1-Mut1 (Fig. 4g). These data demonstrated that HOTAIRM1 directly binds to the EGR1 protein.

**Fig. 5:**
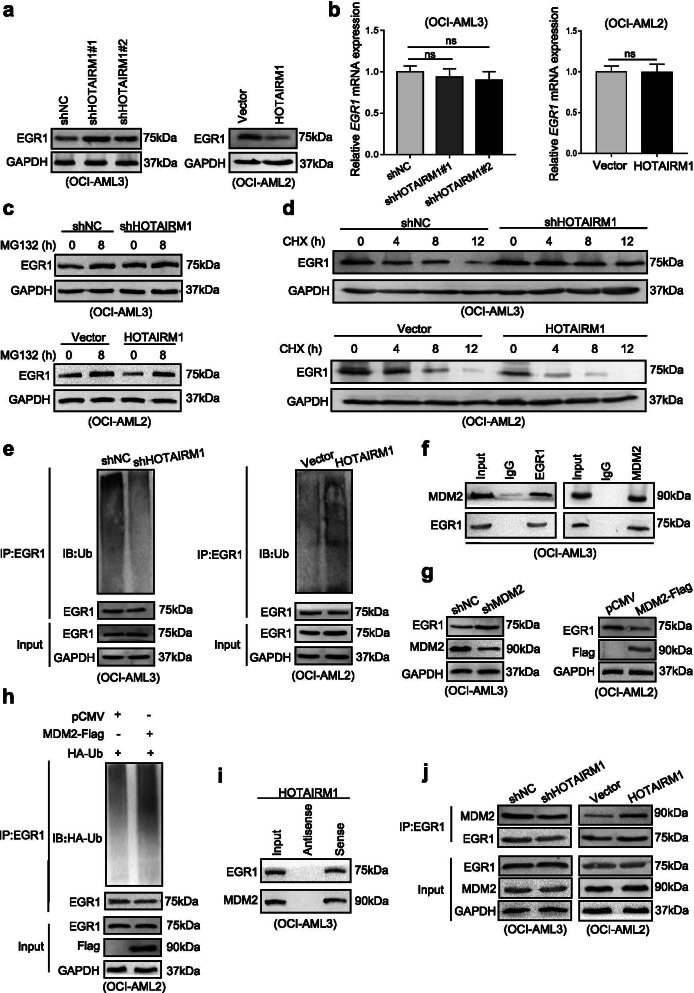
HOTAIRM1 promotes EGR1 degradation through MDM2-mediated ubiquitination. **a** Western blot analysis of EGR1 levels in the different transfection groups of OCI-AML3 and OCI-AML2 cells. **b** Relative EGR1 mRNA expression in HOTAIRM1-silenced and HOTAIRM1-overexpressing cells. **c** EGR1 protein expression in transfected OCI-AML3 and OCI-AML2 cells after treatment with MG132. **d** EGR1 expression in transfected OCI-AML3 and OCI-AML2 cells treated with CHX. **e** Western blot analysis of endogenous EGR1 ubiquitination in transfected OCI-AML3 and OCI-AML2 cells. **f** IP assays were used to investigate the binding of MDM2 to EGR1 in OCI-AML3 cells. **g** The level of EGR1 and MDM2 were examined by western blotting in OCI-AML3 and OCI-AML2 cells transduced with shMDM2 or transfected with the MDM2-Flag plasmid. **h** Western blot analysis of EGR1 ubiquitination in OCI-AML2 cells transfected with the MDM2-Flag plasmid. **i** The interactions between EGR1 or MDM2 and HOTAIRM1 were validated by RNA pulldown and subsequent western blotting. **j** The binding of MDM2 to EGR1 in HOTAIRM1-silenced and HOTAIRM1-overexpressing cells was investigated by IP assays. The data are presented as the mean ± SD of three independent experiments. *P < 0.05, **P < 0.01, ***P < 0.001. n.s. indicates no significant difference

Next, we focused on the biochemical consequences of the interaction between HOTAIRM1 and EGR1. Overexpression of EGR1 (Additional file 16: Figure S11a) had no effect on the HOTAIRM1 level (Additional file 16: Figure S11b). Conversely, HOTAIRM1 expression negatively regulated the EGR1 protein level (Fig. 5a) without altering the EGR1 mRNA level (Fig. 5b). On the basis of these data, we further investigated whether HOTAIRM1 is involved in the regulation of EGR1 protein stability. As shown in Fig. 5c, the proteasome inhibitor MG132 antagonized the decrease the in EGR1 level caused by HOTAIRM1. In addition, treatment with the protein synthesis inhibitor CHX demonstrated that HOTAIRM1 decreased the half-life of the EGR1 protein (Fig. 5d). Subsequent ubiquitination assays revealed that HOTAIRM1 knockdown decreased the level of ubiquitinated EGR1 protein, whereas HOTAIRM1 overexpression increased EGR1 ubiquitination (Fig. 5e). These findings suggested that HOTAIRM1 reduces the stability of EGR1 by promoting its ubiquitination.

Later, we identified which ubiquitin ligase might target EGR1 for degradation in leukemia cells. Murine double minute 2 (MDM2), a well-known E3 ubiquitin-protein ligase, was predicted to interact with EGR1 through bioinformatics analysis (UbiBrowser) (Additional file 17: Figure S12a-b). Indeed, we detected the interaction of endogenous MDM2 and EGR1 in OCI-AML3 cells by IP (Fig. 5f). Importantly, knockdown of MDM2 increased but overexpression of MDM2 decreased the EGR1 protein levels (Fig. 5g). We further confirmed that MDM2 increased EGR1 ubiquitination (Fig. 5h). Finally, we examined the role of HOTAIRM1 in MDM2-mediated EGR1 degradation. The association of HOTAIRM1 with both EGR1 and MDM2 was verified by RNA pulldown assays (Fig. 5i). Correspondingly, IP assays showed that downregulation of HOTAIRM1 inhibited the interaction between EGR1 and MDM2, whereas HOTAIRM1 overexpression had the opposite effect (Fig. 5j). Considering these results collectively, we concluded that HOTAIRM1 promotes EGR1 degradation through MDM2-mediated ubiquitination.

### Nuclear HOTAIRM1 promotes autophagy and proliferation by downregulating EGR1 expression

**Fig. 6:**
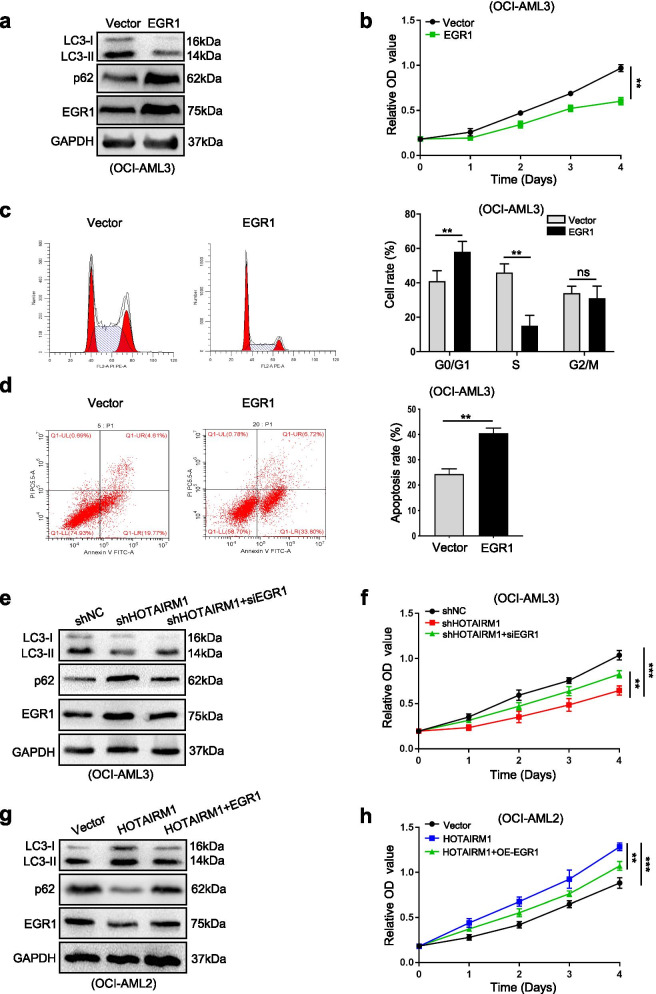
Nuclear HOTAIRM1 promotes leukemia cell autophagy and proliferation through EGR1. **a** Western blot analysis of LC3-II, p62 and EGR1 levels in OCI-AML3 cells transduced with the EGR1 expression vector. **b** CCK-8 assays were applied to test the proliferation of transfected OCI-AML3 cells. **c-d** Flow cytometry was performed to assess the cell cycle (**c**) and apoptosis (**d**) in transfected OCI-AML3 cells. **e, g** Western blot analysis of LC3-II, p62 and EGR1 levels in HOTAIRM1-silenced OCI-AML3 cells after EGR1 knockdown (**e**) and in HOTAIRM1-overexpressing OCI-AML2 cells after EGR1 overexpression (**g**). **f, h** CCK-8 assays were used to analyze cell proliferation in transfected OCI-AML3 (**f**) and OCI-AML2 cells (**h**). The data are presented as the mean ± SD of three independent experiments. *P < 0.05, **P < 0.01, ***P < 0.001. n.s. indicates no significant difference

EGR1 has been linked to tumor suppression during cancer progression [30]. However, the role of EGR1 in leukemia cells is still unclear. We found that forced expression of EGR1 significantly inhibited autophagy (Fig. 6a), proliferation ((Fig. 6b) and cell cycle progression (Fig. 6c) but resulted in a significant increase in apoptosis (Fig. 6d). Furthermore, to determine whether HOTAIRM1 regulates leukemia cell autophagy and proliferation via EGR1, we performed rescue experiments. Silencing EGR1 partially reversed the HOTAIRM1 knockdown-induced reductions in autophagy (Fig. 6e) and proliferation (Fig. 6f, Additional file 18: Figure 13a-b). Conversely, EGR1 overexpression partially reversed the promotive effects of HOTAIRM1 on autophagy (Fig. 6g) and proliferation (Fig. 6h, Additional file 18: Figure 13c-d). Taken together, these data revealed that nuclear HOTAIRM1 promotes autophagy and proliferation by downregulating EGR1.

### Cytoplasmic HOTAIRM1 promotes autophagy and proliferation by sponging miR-152-3p to upregulate ULK3 expression

**Fig. 7:**
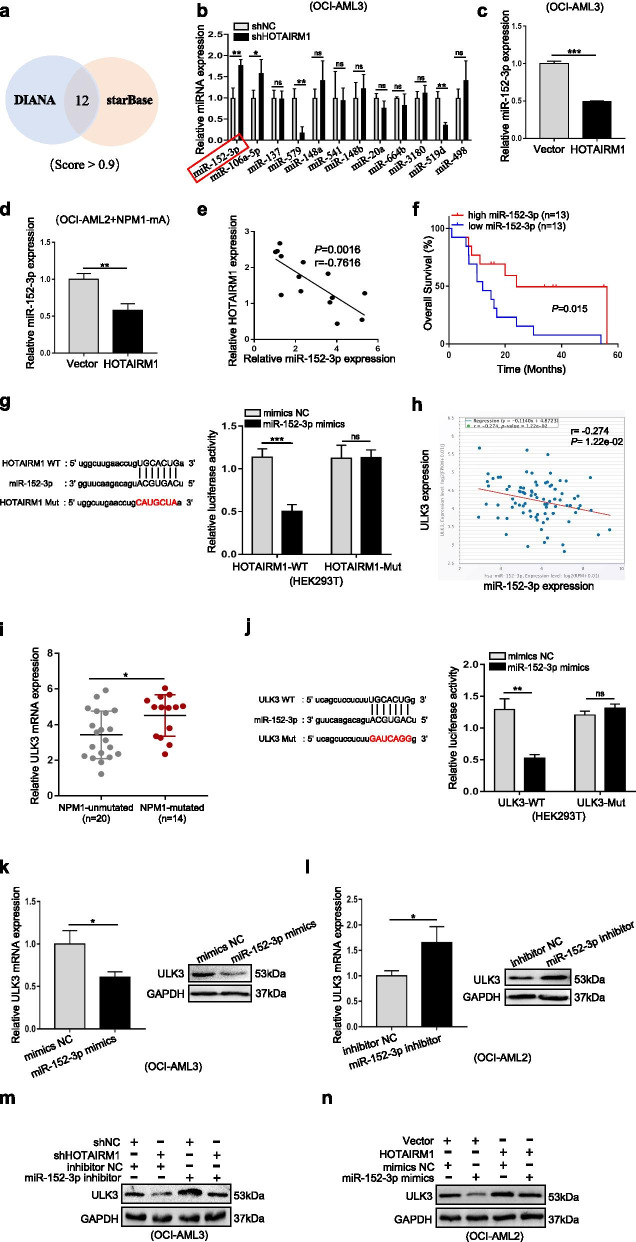
Cytoplasmic HOTAIRM1 regulates the miR-152-3p/ULK3 axis to promote leukemia cell autophagy and proliferation. **a** Venn diagram showing the number of common miRNAs (score > 0.9) targeting the HOTAIRM1 3’UTR predicted by two bioinformatics software tools. **b** Relative levels of candidate miRNAs in OCI-AML3 cells transfected with shHOTAIRM1. **c-d** miR-152-3p expression levels in OCI-AML3 (**c**) and OCI-AML2 + NPM1-mA (**d**) cells transfected with the HOTAIRM1 expression vector. **e** Correlation of HOTAIRM1 expression with miR-152-3p expression in 14 NPM1-mutated AML samples. **f** Survival analysis based on miR-152-3p expression in NPM1-mutated AML samples using TCGA data. **g** Luciferase reporter assays were used to detect luciferase activity in HOTAIRM1-WT/Mut-transfected HEK293T. **h** The correlation between miR-152-3p and ULK3 RNA expression levels was analyzed in the TCGA AML dataset by using starBase. **i** Expression level of ULK3 in primary AML blasts without (*n* = 20) and with NPM1 mutation (*n* = 14). **j** Luciferase reporter assays were used to detect luciferase activity in ULK3-WT/Mut-transfected HEK293T. **k-l** Relative ULK3 mRNA and protein expression in OCI-AML3 cells after transfection with the miR-152-3p mimic (**k**) and in OCI-AML2 cells after transfection with the miR-152-3p inhibitor (**l**). **m-n** Western blot analysis of ULK3 levels in the different transfection groups of HOTAIRM1-silenced OCI-AML3 cells after miR-152-3p downregulation (**m**) and in HOTAIRM1-overexpressing OCI-AML2 cells after miR-152-3p upregulation (**n**). The data are presented as the mean ± SD of three independent experiments. *P < 0.05, **P < 0.01, ***P < 0.001. n.s. indicates no significant difference

Given that HOTAIRM1 was also expressed in the cytoplasm, we then explored the role of cytoplasmic HOTAIRM1 in NPM1-mutated AML cells. HOTAIRM1 has already been identified as a microRNA sponge, including in AML [31, 32]. In this study, we observed that Ago2, the core component of the RNA-induced silencing complex, bound to HOTAIRM1 (Fig. 4c-d). A total of 12 miRNAs binding to HOTAIRM1 were identified by intersecting the predictions of lncRNA target prediction bioinformatics software tools (DIANA and starBase) (Fig. 7a), and miR-152-3p was found to exhibit the greatest change in expression upon HOTAIRM1 modulation (Fig. 7b-c). Next, the negative correlation between HOTAIRM1 and miR-152-3p expression in NPM1-mutated cell lines (Fig. 7d) and patients with NPM1-mutated AML (Fig. 7e) was verified by qRT-PCR. In addition, we analyzed the association between miR-152-3p expression and overall survival in patients with NPM1-mutated AML through the TCGA database and found that low expression of miR-152-3p was associated with poor overall survival in patients (Fig. 7f). Furthermore, luciferase reporter assays revealed that HOTAIRM1-WT-transfected cells showed low luciferase activity in the presence of miR-152-3p, whereas HOTAIRM1-Mut-transfected cells did not show a significant response to miR-152-3p (Fig. 7g). These findings clarified that HOTAIRM1 directly targets miR-152-3p.

Later, we investigated the putative target genes of miR-152-3p and found a trend toward a negative correlation between miR-152-3p and unc-51-like kinase 3 (ULK3) expression in the TCGA AML dataset (Fig. 7h). ULK3 is a kinase that induces autophagy [33–35] and was found to be highly expressed in NPM1-mutant AML (Fig. 7i). Importantly, miR-152-3p mimics significantly attenuated the luciferase activity of ULK3-WT (Fig. 7j). Moreover, miR-152-3p upregulation led to decreased ULK3 mRNA and protein levels (Fig. 7k), and the opposite results were found for miR-152-3p downregulation (Fig. 7l). Furthermore, ULK3 expression was downregulated by HOTAIRM1 knockdown, while simultaneous miR-152-3p knockdown reversed the inhibition of ULK3 induced by HOTAIRM1 knockdown in OCI-AML3 cells (Fig. 7m). In contrast, the promotion of ULK3 expression by HOTAIRM1 upregulation was alleviated after miR-152-3p overexpression (Fig. 7n). Finally, forced expression of ULK3 reversed the inhibition of autophagy and proliferation (Additional file 19: Figure S14a-b) and the increase in apoptosis (Additional file 20: Figure S15a-b) induced by HOTAIRM1 knockdown. On the other hand, ULK3 silencing reversed the autophagy-enhancing, cell proliferation-promoting (Additional file 19: Figure S14c-d) and apoptosis-inhibiting (Additional file 20: Figure S15c-d) effects of HOTAIRM1 upregulation. Taken together, these results implied that cytoplasmic HOTAIRM1 promotes autophagy and proliferation by sponging miR-152-3p to upregulate ULK3 expression.

### HOTAIRM1 knockdown impairs leukemogenesis in vivo

**Fig. 8:**
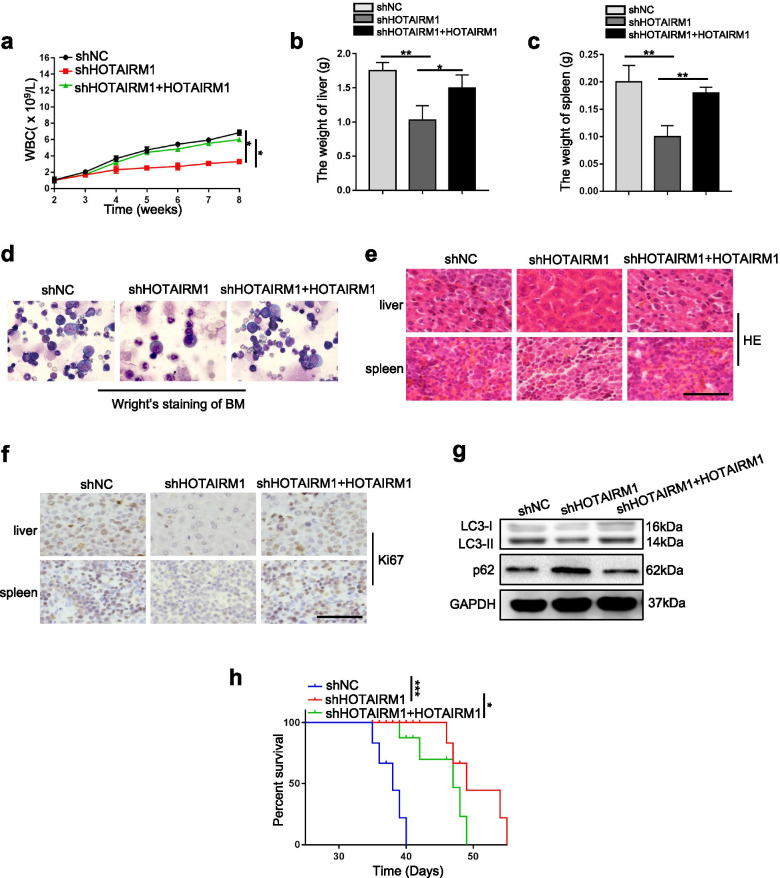
HOTAIRM1 knockdown impairs leukemogenesis in vivo. **a** Blood was collected from all animals via the tail vein 4 weeks after cell transplantation, and the total WBC count was determined. **b-c** The mean liver (**b**) and spleen (**c**) weights of mice transplanted with shNC, shHOTAIRM1 and shHOTAIRM1 + HOTAIRM1 cells were quantified. **d** Immature cells from bone marrow were evaluated by Wright’s staining. **e** Representative images of histologic sections of livers and spleens from mice transplanted with cells treated as indicated. Scale bar: 50 μm. **f** IHC staining of Ki-67 in liver and spleen tissue from mice transplanted with cells. Scale bar: 50 μm. **g** The protein levels of LC3 and p62 in each group were determined by western blotting. **h** Kaplan-Meier survival curves of mice in each group

Based on our in vitro findings regarding HOTAIRM1, we speculated that HOTAIRM1 might play an important role in leukemogenesis in vivo. A xenograft model was established in NOD/SCID mice by injection of engineered OCI-AML3 cells (shNC, shHOTAIRM1 and shHOTAIRM1 + HOTAIRM1). As shown in Fig. 8a-c, HOTAIRM1 knockdown led to lower white blood cell (WBC) counts and liver and spleen weights. Wright’s staining of bone marrow smears showed that HOTAIRM1 knockdown significantly reduced the number of leukemia cells (Fig. 8d). In addition, HE staining revealed that fewer leukemia cells had infiltrated the liver and spleen in the shHOTAIRM1 group (Fig. 8e). IHC staining of tissues infiltrated by leukemia cells with anti-Ki-67 antibodies further implied that knockdown of HOTAIRM1 can inhibit leukemia cell proliferation (Fig. 8f). Western blot analysis showed that HOTAIRM1 knockdown decreased the LC3-II level and increased the p62 level (Fig. 8g). Moreover, the survival of mice implanted with shHOTAIRM1 cells was significantly prolonged compared with that of mice implanted with shNC cells (Fig. 8h). Notably, forced expression of HOTAIRM1 reversed the inhibitory effects of HOTAIRM1 knockdown on leukemogenesis (Fig. 8a-h). In conclusion, these data suggested that HOTAIRM1 knockdown impairs the progression of NPM1-mutated AML.

## Discussion

**Fig. 9:**
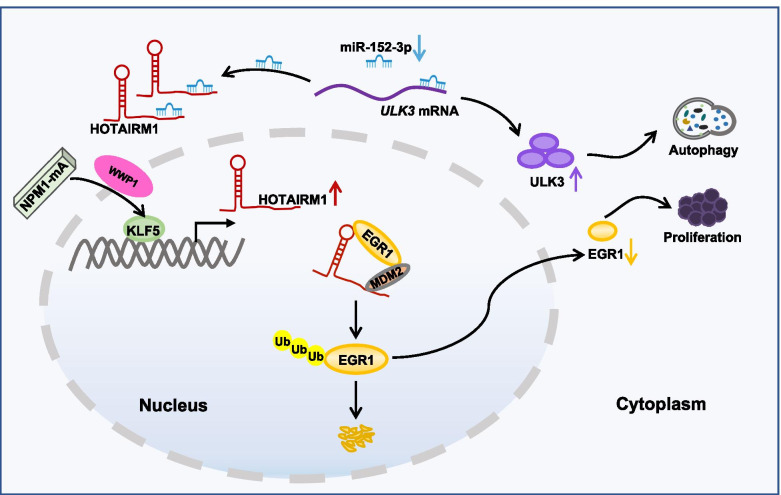
Schematic diagram describing the functional significance of HOTAIRM1 in NPM1-mutated leukemia cells. HOTAIRM1 expression was induced by mutant NPM1 via KLF5-dependent transcriptional regulation. Nuclear HOTAIRM1 promoted EGR1 degradation by enhancing the MDM2-EGR1 interaction, while cytoplasmic HOTAIRM1 increased ULK3 expression by competitively sponging miR-152-3p, therefore contributing to autophagy and proliferation in NPM1-mutated AML cells

Accumulating evidence implies that aberrant expression of lncRNAs plays a critical role in leukemia initiation and prognostic outcomes [36–38]. However, only a very small number of lncRNAs have been experimentally validated and functionally annotated in NPM1-mutated AML [22, 39, 40]. Our data herein demonstrated the high level of lncRNA HOTAIRM1 in NPM1-mutated leukemia cells and indicated that HOTAIRM1 was upregulated at least partially by mutant NPM1 via KLF5-dependent transcriptional regulation. Furthermore, nuclear HOTAIRM1 promoted EGR1 ubiquitination by enhancing the MDM2-EGR1 interaction, while cytoplasmic HOTAIRM1 increased ULK3 expression by competitively sponging miR-152-3p, therefore contributing to leukemia cell autophagy and proliferation (Fig. 9).

In the current study, we analyzed gene expression profiles using public databases and found that HOTAIRM1, a lncRNA transcribed from the antisense strand of the HOXA gene [20], was one of the most significantly upregulated lncRNAs. High expression of HOTAIRM1 was subsequently confirmed by qRT-PCR in both NPM1-mA-positive OCI-AML3 cells and primary AML blasts. In addition, our findings were supported by those of a previous study indicating that HOTAIRM1 expression was elevated in myeloid leukemia cells [18, 21]. Additionally, other HOX cluster lncRNAs, such as HOXA10-AS and HOXB-AS3, have been reported to be significantly overexpressed in NPM1-mutated leukemia cells [39, 41]. Deregulated lncRNAs have prognostic applications in AML [42]. Our results derived from public databases showed that a high level of HOTAIRM1 indicated a poorer prognosis in NPM1-mutated AML, consistent with a previous study that established that patients with NPM1-mutated AML with high HOTAIRM1 expression have worse prognoses [21]. Indeed, the clinical prognostic role of HOTAIRM1 in patients with NPM1-mutated AML awaits further study.

Next, we focused on the molecular mechanism underlying the high expression of HOTAIRM1 in NPM1-mutated AML. Recently, Gourvest M et al. revealed that the localization of the lncRNA LONA is dependent on the NPM1 mutational status [22]. Here, the cellular localization of HOTAIRM1 in leukemia cell lines remained the same regardless of the mutational status of NPM1. Notably, NPM1-mA significantly increased the HOTAIRM1 level and the luciferase activity of the HOTAIRM1 promoter. We next sought to identify the specific transcription factors involved in this process. Bioinformatics analysis and mechanistic experiments suggested that KLF5 directly activated the transcription of HOTAIRM1 by functioning as a transcriptional activator. More importantly, NPM1-mA knockdown inhibited KLF5-mediated transcriptional activation of HOTAIRM1. In addition, we investigated the mechanism by which NPM1 regulates KLF5 transcriptional activity and found that NPM1-mA weakened the KLF5-WWP1 interaction, thereby diminishing WWP1-mediated ubiquitination and degradation of KLF5. Collectively, our data revealed that the mutant form of NPM1 is responsible for the upregulation of HOTAIRM1 in AML cells via stabilization of the KLF5 transcription factor. Interestingly, lncRNAs have also been reported to regulate NPM1c + −mediated transcription. Zhu et al. [40] reported that the lncRNA HOXBLINC loss attenuates NPM1c + −driven leukemogenesis by rectifying the signature of NPM1c + leukemic transcription programs. In addition, lncRNA expression is susceptible to diverse factors at different levels. For example, epidermal growth factor rapidly attenuates the expression of the lncRNA LIMT by enhancing histone deacetylation in its promoter region [43]. N6-methyladenosine (m6A) methylation mediated by METTL3 and METTL14 contributes to the increased stability of LNCAROD [44]. A recent study described that the DNA methyltransferase DNMT3b can reduce HOTAIRM1 expression in AML [45]. Thus, other possible regulatory mechanisms contributing to HOTAIRM1 expression in NPM1-mutated AML merit further exploration.

Next, we explored the biological effects of high HOTAIRM1 expression in NPM1-mutated leukemia. Our previous work showed high autophagic activity in NPM1-mutated AML cells [46]. Therefore, we first investigated the role of HOTAIRM1 in regulating autophagy and found that HOTAIRM1 elevated autophagic activity. Accumulating evidence suggests that the functional relationship among autophagy, proliferation and apoptosis is complex [47]. Here, we observed that HOTAIRM1 promoted cell proliferation and inhibited apoptosis. In vivo experiments further suggested that HOTAIRM1 promoted the malignant phenotype of NPM1-mutated AML. Collectively, these findings implied that HOTAIRM1 might play an oncogenic role in NPM1-mutated AML. Our idea was supported by a research showing that knockdown of HOTAIRM1 facilitated Ara-C-induced apoptosis in the AML cell lines HL-60 and THP-1 [48]. Notably, Chen et al. [31] reported that HOTAIRM1 plays a role as a tumor suppressor gene in acute promyelocytic leukemia (APL). In that study, HOTAIRM1 enhanced the autophagy pathway, which promoted ATRA-induced degradation of the PML-RARA oncoprotein and resulted in cell differentiation. These findings and our data help to identify HOTAIRM1 as a general regulator of autophagy and survival processes in leukemia cells.

To investigate the potential mechanisms by which HOTAIRM1 contributes to the malignant phenotypes of NPM1-mutated leukemia cells, we examined its subcellular localization. HOTAIRM1 was localized in both the nucleus and cytoplasm. We first tested the role of nuclear HOTAIRM1 in leukemia cells. EGR1, a Cys2-His2-type zinc finger transcription factor, was identified as a specific interacting partner of HOTAIRM1. Further research showed that the proteasome pathway contributed to HOTAIRM1-induced EGR1 protein degradation. Then, MDM2 was confirmed to target EGR1 for ubiquitin-mediated degradation. More notably, nuclear HOTAIRM1 promoted EGR1 degradation by enhancing the MDM2-EGR1 interaction. Previous studies have suggested that lncRNAs can function as scaffolds to influence protein expression. For example, lnczc3h7a acts as a scaffold to facilitate the interaction between TRIM25 and activated RIG-I, consequently increasing TRIM25-mediated K63-linked ubiquitination of RIG-I [49]. Additionally, the lncRNA CRNDE acts as a scaffold for DMBT1 and C-IAP1 complexes to activate the PI3K-AKT pathway [50]. Moreover, lncRNAs play roles as signals, decoys, and guides for regulatory proteins. LncRNA-Gas5 binds to the DNA-binding domain of the glucocorticoid receptor by acting as a decoy [51]. The lncRNA PACER dissociates the repressive NF-kB subunit p50 from the COX-2 promoter to activate COX-2 gene expression [52]. Recently, a study reported that HOTAIRM1 binds to ILF3 and blocks the effects of ILF3 on stabilizing pre-miR-144 [53]. Thus, whether other HOTAIRM1-associated proteins are involved in NPM1-mutated AML requires further investigation. After investigating the role of nuclear HOTAIRM1, we explored the function of cytoplasmic HOTAIRM1 in leukemia cells. The miR-152-3p level was significantly changed after modulation of HOTAIRM1, and miR-152-3p was found to bind directly to HOTAIRM1. More importantly, miR-152-3p also bound to ULK3 and regulated its expression. In addition, our data identified cytoplasmic HOTAIRM1 as a competing endogenous RNA (ceRNA) for miR-152-3p and antagonized its repression of ULK3 protein translation. These results are similar to the observation recently reported by Lin et al. [54], which indicated that HOTAIRM1 increases ZEB2 expression by sponging miR-873-5p. A recent study described that the lncRNA LBCS interacts with heterogeneous nuclear ribonucleoprotein K (hnRNPK) to suppress androgen receptor (AR) translation efficiency by forming a complex with hnRNPK and AR mRNA [55]. Thus, the other potential mechanisms underlying the oncogenic role of cytoplasmic HOTAIRM1 need to be determined. Most importantly, we are the first to reveal that HOTAIRM1 plays different regulatory roles in the nucleus and cytoplasm in NPM1-mutated AML. Prompted by these findings, we further investigated the role of the downstream HOTAIRM1 target genes EGR1 and ULK3 in leukemia cells. These results revealed that EGR1 and ULK3 participated in the modulation of autophagy and proliferation induced by HOTAIRM1. Our findings were consistent with the implication of EGR1 in the regulation of cell proliferation [56], migration [57] and apoptosis [58]. In addition, ULK3, an ATG1-related isoform [59], is involved in many biological processes, including autophagy [33], cytokinesis [34] and Hedgehog signaling [35]. Therefore, these findings demonstrate that HOTAIRM1 promotes leukemia cell autophagy and proliferation by regulating EGR1 and ULK3 expression.

In summary, the results of this study indicate that HOTAIRM1 functions as an oncogene in NPM1-mutated AML. Upregulation of HOTAIRM1 is induced by mutant NPM1 via KLF5-dependent transcriptional regulation. Importantly, high expression of HOTAIRM1 promotes autophagy and proliferation via the functions of HOTAIRM1 as a scaffold to recruit MDM2 to EGR1 in the nucleus and as a sponge for miR-152-3p to upregulate ULK3 in the cytoplasm. These findings shed new light on the lncRNAs involved in the pathogenesis of NPM1-mutated AML and indicate that HOTAIRM1 may be an important target for future treatment of this distinct leukemia subtype.

## Supplementary Information

**Additional file 1 **: **Table S1.** Clinical characteristics of newly diagnosed AML patients.

**Additional file 2 **: **Table S2.** Primers used for qRT-PCR.

**Additional file 3 **: **Table S3.** Primers used for ChIP-qPCR.

**Additional file 4 : Table S4.** The top 10 predicted factors bound to HOTAIRM1 promoter by JASPAR database analysis.

**Additional file 5 **: **Table S5.** The top 10 proteins with specific binding to HOTAIRM1 identified by mass spectrometry analysis.

**Additional file 6 **: **Figure S1.** Stable NPM1-mA-GFP expressing OCI-AML2 cells (OCI-AML2 + NPM1-mA) were generated. a Cytoplasmic localization of NPM1-mA in the OCI-AML2 stably expressing Flag-GFP tagged NPM1-mA. Cytospun cells were fixed and stained with DAPI. b The level of NPM1-mA in leukemia cells were measured by western blot analysis.

**Additional file 7 **: **Figure S2.** The expression levels of HOTAIRM1 in the nucleus and cytoplasm of leukemia cells were measured by qRT-PCR.

**Additional file 8 **: **Figure S3.** NPM1-mA expression in leukemia cells in the different treatment groups. a The efficiency of RNA interference-mediated knockdown of NPM1-mA expression was determined by qRT-PCR. b The efficiency of RNA interference-mediated knockdown of NPM1-mA expression was determined by western blotting. c NPM1-mA protein levels in NPM1-mA enforced OCI-AML2 cells. d Western blot analysis of NPM1-mA in OCI-AML3 cells treated with KPT-330. β-tubulin as the cytoplasmic control and PCNA as the nuclear control. The data are presented as the mean ± SD of three independent experiments.

**Additional file 9 **: **Figure S4.** ChIP-qPCR analysis of NPM1 occupancy on the HOTAIRM1 promoter in leukemia cells.

**Additional file 10 **: **Figure S5.** Enrichment of KLF5 on the E2 fragment of the HOTAIRM1 promoter was measured by ChIP assays in leukemia cells.

**Additional file 11 **: **Figure S6.** Co-IP assays were used to evaluate the binding of NPM1 and KLF5, NPM1-wt and WWP1 in OCI-AML3 cells. a-b The interaction between NPM1-mA (a) or NPM1-wt (b) and KLF5 in OCI-AML3 cells were determined by IP assays. c IP assays were used to determine the binding of WWP1 to NPM1-wt in OCI-AML3 cells.

**Additional file 12 **: **Figure S7.** HOTAIRM1 expression in the different transfected cells. a-b HOTAIRM1 expression in HOTAIRM1-silenced (a) and HOTAIRM1-enforced OCI-AML3 cells (b). c-d HOTAIRM1 expression in HOTAIRM1-silenced (c) as well as HOTAIRM1-enforced OCI-AML2 + NPM1-mA cells (d). e HOTAIRM1 expression in HOTAIRM1-enforced OCI-AML2 cells.

**Additional file 13 **: **Figure S8.** HOTAIRM1 promotes autophagy and proliferation in transfected OCI-AML2 + NPM1-mA leukemia cells. a, i Western blot analysis of LC3-II and p62 levels in OCI-AML2 + NPM1-mA cells after transfection of shHOTAIRM1 (a) and HOTAIRM1 expression vector (i). b, j Representative microscopy images of LC3 puncta in HOTAIRM1-silenced OCI-AML2 + NPM1-mA (b) and HOTAIRM1-enforced OCI-AML2 + NPM1-mA (j). The bar graphs showed the quantification of the fluorescent puncta data. Scale bar: 25 μm. c, k Evaluation of cell viability in OCI-AML2 + NPM1-mA cells transfected with shHOTAIRM1 (c) or HOTAIRM1 plasmid (k) for indicated by CCK-8 assays. d, l Evaluation of cell proliferation in HOTAIRM1-silenced (d) and HOTAIRM1-enforced OCI-AML + NPM1-mA cells (l) for indicated by EdU assays. The bar graphs showed the percentage of EdU positive cells. Scale bar: 100 μm. e, m Flow cytometry was performed to assess cell cycle of HOTAIRM1-silenced (e) and HOTAIRM1-enforced OCI-AML2 + NPM1-mA cells (m). The bar graph shows the percentages of G0/G1-, S-, and G2/M-phase cells. f, n Flow cytometry was used to detect apoptosis of HOTAIRM1-silenced (f) and HOTAIRM1-enforced OCI-AML2 + NPM1-mA cells (n). LL, dead cells; UL, viable cells; LR, early apoptotic cells; UR, late apoptotic cells. g, o Flow cytometric analysis was performed to analyze apoptosis of OCI-AML2 + NPM1-mA cells after transfection with shHOTAIRM1 (g) or HOTAIRM1 plasmid (o), followed by treatment with 200 nM Ara-C for 48 h. h, p The protein levels of Cyclin D1, CDK4, Bax and Bcl-2 in HOweTAIRM1-silenced (h) and HOTAIRM1-enforced OCI-AML2 + NPM1-mA cells (p). The data are presented as the mean ± SD of three independent experiments. **P < 0.01, ***P < 0.001. n.s. indicates no significant difference.

**Additional file 14 **: **Figure S9.** HOTAIRM1 promotes autophagy and proliferation in transfected OCI-AML2 leukemia cells. a Western blot analysis of LC3-II and p62 levels in OCI-AML2 cells after transfection of HOTAIRM1 expression vector. b Representative microscopy images of LC3 puncta in HOTAIRM1-enforced OCI-AML2 cells. The bar graphs showed the quantification of the fluorescent puncta data. Scale bar: 25 μm. c Evaluation of cell viability in OCI-AML2 cells transfected with HOTAIRM1 plasmid for indicated by CCK-8 assays. d Evaluation of cell proliferation in HOTAIRM1-enforced OCI-AML2 cells for indicated by EdU assays. The bar graphs showed the percentage of EdU positive cells. Scale bar: 100 μm. e Flow cytometry was performed to assess cell cycle of HOTAIRM1-enforced OCI-AML2 cells. The bar graph shows the percentages of G0/G1-, S-, and G2/M-phase cells. f Flow cytometry was used to detect apoptosis of HOTAIRM1-enforced OCI-AML2 cells. LL, dead cells; UL, viable cells; LR, early apoptotic cells; UR, late apoptotic cells. g The protein levels of Cyclin D1, CDK4, Bax and Bcl-2 in HOTAIRM1-enforced OCI-AML2 cells. The data are presented as the mean ± SD of three independent experiments. **P < 0.01, ***P < 0.001. n.s. indicates no significant difference.

**Additional file 15 **: **Figure S10.** HOTAIRM1 expression in HOTAIRM1-silenced OCI-AML3 cells transfected with different vectors.

**Additional file 16 **: **Figure S11.** EGR1 does not affect the expression of HOTAIRM1. a The efficiency of EGR1 overexpression in OCI-AML3 cells was validated by western blotting. b qRT-PCR was used to detect the expression of HOTAIRM1 in EGR1-enforced OCI-AML3 cells. The data are presented as the mean ± SD of three independent experiments. n.s. indicates no significant difference.

**Additional file 17 **: **Figure S12.** Screening for the ubiquitin ligase of EGR1. a Network view of the predicted E3 ubiquitin ligase for EGR1. b Recognition motif for the potential E3 ubiquitin ligase MDM2 in the EGR1 sequence.

**Additional file 18 **: **Figure S13.** Nuclear HOTAIRM1 promotes cell cycle progression and inhibits apoptosis in leukemia cells through EGR1. a, c Flow cytometry was used to determine and compare the differences in cell cycle progression in differently transfected groups of OCI-AML3 (a) and OCI-AML2 cells (c). b, d Flow cytometry was used to determine and compare differences in apoptosis in differently transfected groups of OCI-AML3 (b) and OCI-AML2 cells (d). The data are presented as the mean ± SD of three independent experiments. *P < 0.05, **P < 0.01. n.s. indicates no significant difference.

**Additional file 19 **: **Figure S14.** Cytoplasmic HOTAIRM1 promotes leukemia cell autophagy and proliferation through ULK3. a, c Western blot analysis of LC3-II, p62 and ULK3 levels in HOTAIRM1-silenced OCI-AML3 cells following ULK3 overexpression (a) and HOTAIRM1-overexpressed OCI-AML2 cells following ULK3 knockdown (c). b, d CCK-8 assays were used to analyze cell proliferation in transfected OCI-AML3 (b) and OCI-AML2 cells (d). The data are presented as the mean ± SD of three independent experiments. *P < 0.05, **P < 0.01, ***P < 0.001. n.s. indicates no significant difference.

**Additional file 20 **: **Figure S15.** Cytoplasmic HOTAIRM1 promotes cell cycle progression and inhibits apoptosis in leukemia cells through ULK3. a-b Flow cytometry was used to analyze the changes in cell the cycle (a) and apoptosis (b) in HOTAIRM1-silenced OCI-AML3 cells following ULK3 upregulation. c-d Flow cytometry was used to analyze the change in the cell cycle (c) and apoptosis (d) in HOTAIRM1-overexpressed OCI-AML2 cells following ULK3 downregulation. The data are presented as the mean ± SD of three independent experiments. *P < 0.05, **P < 0.01, ***P < 0.001. n.s. indicates no significant difference.

## Data Availability

All data generated or analysed during this study are included in this published article [and its supplementary information files].

## Abbreviations

AML: Acute myeloid leukemia
WHO: World Health Organization
NPM1: Nucleophosmin
NPM1c +: Cytoplasmic mutant NPM1
CRM1: Chromosome maintenance region 1
ATRA: All-trans retinoic acid
ATO: Arsenic trioxide
LncRNA: Long noncoding RNA
ceRNA: Competing endogenous RNA
HOTAIRM1: HOX antisense intergenic RNA myeloid 1
Ara-C: Cytarabine
KLF5: Kruppel like factor 5
WWP1: WW Domain-Containing Protein 1
MDM2: Murine double minute 2
EGR1: Early growth response protein 1
ULK3: Unc-51 like kinase 3
GEO: Gene Expression Omnibus
TCGA: The Cancer Genome Atlas
OS: Overall survival
DSMZ: Deutsche Sammlung von Mikroorganismen und Zellkulturen GmbH
NPM1-mA: NPM1 mutation type A
ATCC: American Type Culture Collection
FBS: Fetal bovine serum
qRT-PCR: Quantitative real-time PCR
shRNA: Lentivirus-based short hairpin RNA
siRNA: Short interfering RNA
SDS-PAGE: Sodium dodecyl sulfate polyacrylamide gel electrophoresis
PVDF: Polyvinylidene fluoride
Ago2: Argonaute 2
CDK4: Cyclin-dependent kinase 4
LC3: Microtubule-associated protein 1 light chain 3
CHX: Cycloheximide
ChIP: Chromatin immunoprecipitation
CCK-8: Cell Counting Kit-8
EdU: 5-ethynyl-2′-deoxyuridine
FISH: Subcellular fractionation and fluorescence in situ hybridization
RIP: RNA immunoprecipitation
NC: Negative control
IP: Immunoprecipitation
WBC: White blood counts
HE: Hematoxylin and eosin
IHC: Immunohistochemistry
SD: Standard deviation
hnRNPK: Heterogeneous nuclear ribonucleoprotein K
AR: Androgen receptor
