# Supplementary material for: Mutant NPM1-regulated lncRNA HOTAIRM1 promotes leukemia cell autophagy and proliferation by targeting EGR1 and ULK3
Source: J Exp Clin Cancer Res. 2021 Oct 6;40:312. doi: 10.1186/s13046-021-02122-2 (PMC8493742; doi:10.1186/s13046-021-02122-2)
Supplement: Supplementary file 1 — Additional file 1 : Table S1. Clinical characteristics of newly diagnosed AML patients. [file 13046_2021_2122_MOESM1_ESM.docx]

**Additional file 1: Table S1. Clinical characteristics of newly diagnosed AML patients**

| **Characteristics** | **Median (range)** | **No. of cases** |
| --- | --- | --- |
| Sex |  |  |
| Female |  | 18 |
| Male |  | 16 |
| Total |  | 34 |
| Median age (years) | 49.5 (15-79) |  |
| Younger than 40 y |  | 10 |
| 40-60 y |  | 13 |
| Older than 60 y |  | 11 |
| Median WBC, 10^9^/L | 62.7 (0.22-347.0) |  |
| Median platelets, 10^9^/L | 60.5 (2.0 - 300.0) |  |
| AML FAB subtype |  |  |
| AML without maturation: M1 |  | 1 |
| AML with maturation: M2 |  | 5 |
| Acute promyelocytic leukemia: M3 |  | 2 |
| Acute myelomonocytic leukemia: M4 |  | 7 |
| Acute monoblastic or monocytic leukemia: M5 |  | 17 |
| Other subtypes |  | 2 |
| Karyotype |  |  |
| Normal |  | 18 |
| t(8;21) |  | 3 |
| t(15;17) |  | 5 |
| inv(16) |  | 2 |
| Unknown |  | 6 |
| Gene mutations |  |  |
| ***NPM1*** |  | **14** |
| *FLT3-ITD* |  | 8 |
| IDH1/IDH2 |  | 6 |
| *DNMT3A* |  | 7 |
| *WT1* |  | 12 |

Abbreviations: AML, acute myeloid leukemia; y, year old; WBC, white blood cell; FAB classification, French-American-British classification, a classification of acute leukemia produced by three-nation joint collaboration.
