# Supplementary material for: Mutant NPM1-regulated lncRNA HOTAIRM1 promotes leukemia cell autophagy and proliferation by targeting EGR1 and ULK3
Source: J Exp Clin Cancer Res. 2021 Oct 6;40:312. doi: 10.1186/s13046-021-02122-2 (PMC8493742; doi:10.1186/s13046-021-02122-2)
Supplement: Supplementary file 2 — Additional file 2 : Table S2. Primers used for qRT-PCR. [file 13046_2021_2122_MOESM2_ESM.docx]

**Additional file 2: Table S2. Primers used for qRT-PCR**

| **Genes** | **Sequences (5' - 3')** |
| --- | --- |
| HOTAIRM1 | F: 5'-TAGTTATTGACCTGGAGACTGGTA-3' |
| NPM1-mA | R: 5'-TCAGTGCACAGGTTCAAGCC-3'  F: 5'-TGGAGGTGGTAGCAAGGTTC-3' |
|  | R: 5'-CTTCCTCCACTGC CAGACAGA-3' |
| NPM1-wt | F: 5'-ACGGTCAGTTTAGGGGCTG-3' |
|  | R: 5'-CTGTGGAACCTTGCTACCACC-3' |
| KLF5 | F: 5′-GATCTAGATATGCCCAGTTC-3′ |
|  | R: 5′-CAGCCTTCCCAGGTACACTTG-3′ |
| EGR1 | F: 5'-GGTCAGTGGCCTAGTGAGC-3'  R: 5'-GTGCCGCTGAGTAAATGGGA-3' |
| ULK3 | F: 5'-GAAGGACACTCGTGAAGTGGT-3' |
|  | R: 5'-CTTGACCAACTCGCTCATGTTA-3' |
| miR-152-3p | F: 5'-CGCGTCAGTGCATGACAGA-3' |
|  | R: 5'-AGTGCAGGGTCCGAGGTATT-3' |
| miR-106a-5p | F: 5'- CGCGAAAAGTGCTTACAGTGC-3' |
|  | R: 5'-AGTGCAGGGTCCGAGGTATT-3' |
| miR-137 | F: 5'- CGCGCGTTATTGCTTAAGAATAC-3' |
|  | R: 5'- AGTGCAGGGTCCGAGGTATT-3' |
| miR-579 | F: 5'- CGCGTCAGTGCATCACAGAA-3' |
|  | R: 5'-AGTGCAGGGTCCGAGGTATT-3' |
| miR-148a | F: 5'- GCGCGTTCATTTGGTATAAACC -3' |
|  | R: 5'-AGTGCAGGGTCCGAGGTATT-3' |
| miR-541 | F: 5'- CGAAAGGATTCTGCTGTCGGT-3' |
|  | R: 5'-AGTGCAGGGTCCGAGGTATT-3' |
| miR-148b | F: 5'-GCGCGTCAGTGCACTACAGAA-3' |
|  | R: 5'-AGTGCAGGGTCCGAGGTATT-3' |
| miR-20a | F: 5'- GCGCGTAAAGTGCTTATAGTGC-3' |
|  | R: 5'-AGTGCAGGGTCCGAGGTATT-3' |
| miR-664b | F: 5'-GCGTTCATTTGCCTCCCAG-3' |
|  | R: 5'-AGTGCAGGGTCCGAGGTATT-3' |
| miR-3180 | F: 5'-TGGGGCGGAGCTTCCG-3' |
|  | R: 5'-AGTGCAGGGTCCGAGGTATT-3' |
| miR-519d | F: 5'-CGCAAAGTGCCTCCCTTT-3' |
|  | R: 5'- AGTGCAGGGTCCGAGGTATT-3' |
| miR-498 | F: 5'-CAGTTTCAAGCCAGGGGGCG-3' |
|  | R: 5'-ATCCAGTGCAGGGTCCGAGG-3' |
| GAPDH | F: 5'-AGCAAGAGCACAAGAGGAAG-3' |
| U6 | R: 5'-GGTTGAGCACAGGGTACTTT-3'  F: 5'-CGGGTTCTCCAAAAGAAAGCA-3' |
|  | R: 5'-CAGCCACAAAAGAGCACAAT-3' |

Abbreviations: F stands for forward; R stands for reverse.
