# Supplementary material for: Mutant NPM1-regulated lncRNA HOTAIRM1 promotes leukemia cell autophagy and proliferation by targeting EGR1 and ULK3
Source: J Exp Clin Cancer Res. 2021 Oct 6;40:312. doi: 10.1186/s13046-021-02122-2 (PMC8493742; doi:10.1186/s13046-021-02122-2)
Supplement: Supplementary file 3 — Additional file 3 : Table S3. Primers used for ChIP-qPCR. [file 13046_2021_2122_MOESM3_ESM.docx]

**Additional file 3: Table S3**. **Primers used for ChIP-qPCR**

| **Genes** | **Sequences (5’- 3’)** |
| --- | --- |
| HOTAIRM1-promoter  HOTAIRM1-Site E1  HOTAIRM1-Site E2 | F: 5'-GGAAGCCATGAGACGGAAATGTAA-3'  R: 5'-CACTTTACACCTCATCACTCACCC-3'  F: 5'-AGGTCATCTGAAAGGAACAGTAGG-3  R: 5'-CTACCTCAGACACTCTGACTACCT-3'  F: 5'-AGGGTGAGTGATGAGGTGTAAAGT-3  R: 5'-CAAAGAAGTCTCAGGGATTGGTGT-3' |

Abbreviations: F stands for forward; R stands for reverse.
