# Supplementary material for: Mutant NPM1-regulated lncRNA HOTAIRM1 promotes leukemia cell autophagy and proliferation by targeting EGR1 and ULK3
Source: J Exp Clin Cancer Res. 2021 Oct 6;40:312. doi: 10.1186/s13046-021-02122-2 (PMC8493742; doi:10.1186/s13046-021-02122-2)
Supplement: Supplementary file 4 — Additional file 4 : Table S4. The top 10 predicted factors bound to HOTAIRM1 promoter by JASPAR database analysis. [file 13046_2021_2122_MOESM4_ESM.docx]

**Additional file 4: Table S4. The top 10 predicted factors bound to HOTAIRM1 promoter by JASPAR database analysis**

| **Matrix ID Name** | **Score** | |
| --- | --- | --- |
| MA0663.1 MLX | | 10.9477 |
| MA1525.1 NFATC4 | | 10.925 |
| **MA0599.1 KLF5** | | **10.7996** |
| MA0105.1 NFKB1 | | 10.7893 |
| MA0039.4 KLF4 | | 10.7812 |
| MA0058.3 MAX | | 10.7667 |
| MA1513.1 KLF15 | | 10.7438 |
| MA1639.1 MEIS1 | | 10.6504 |
| MA0495.1 MAFF | | 10.6486 |
| MA1530.1 NKX6-3 | | 10.6433 |
