# Supplementary material for: Mutant NPM1-regulated lncRNA HOTAIRM1 promotes leukemia cell autophagy and proliferation by targeting EGR1 and ULK3
Source: J Exp Clin Cancer Res. 2021 Oct 6;40:312. doi: 10.1186/s13046-021-02122-2 (PMC8493742; doi:10.1186/s13046-021-02122-2)
Supplement: Supplementary file 5 — Additional file 5 : Table S5. The top 10 proteins with specific binding to HOTAIRM1 identified by mass spectrometry analysis. [file 13046_2021_2122_MOESM5_ESM.docx]

**Additional file 5: Table S5. The top 10 proteins with specific binding to HOTAIRM1 identified by mass spectrometry analysis**

| **Unnsed Acc** | **Coverage(%)** | **#Peptide** | |
| --- | --- | --- | --- |
| 40.87 ACTG | 54. 12999988 | | 23 |
| 34.43 HS90B | 25.40999949 | | 18 |
| 34.12 ENOA | 40.7799989 | | 16 |
| 32.67 ALDOA | 38.19000125 | | 17 |
| **29.2 EGR1** | **24.59000051** | | **14** |
| 27.71 PGK1 | 41.49000049 | | 14 |
| 26.02 CH60 | 27.2300005 | | 13 |
| 25.75 TBA1A | 33.70000124 | | 14 |
| 25.21 G3P | 40.90000093 | | 13 |
| 24.21 TBB5 | 30.63000143 | | 12 |
