# Supplementary material for: Mutant NPM1-regulated lncRNA HOTAIRM1 promotes leukemia cell autophagy and proliferation by targeting EGR1 and ULK3
Source: J Exp Clin Cancer Res. 2021 Oct 6;40:312. doi: 10.1186/s13046-021-02122-2 (PMC8493742; doi:10.1186/s13046-021-02122-2)

**Additional file 6: Figure S1.** Stable NPM1-mA-GFP expressing OCI-AML2 cells (OCI-AML2+NPM1-mA) were generated

**a**

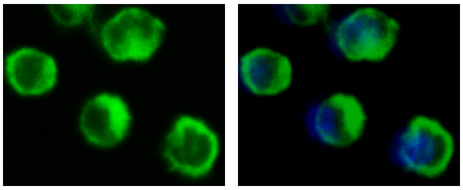

（OCI-AML2+NPM1-mA）

**b**

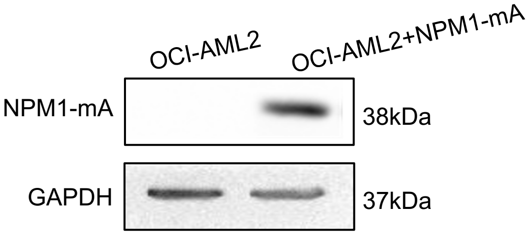

Supplement: Supplementary file 6 — Additional file 6 : Figure S1. Stable NPM1-mA-GFP expressing OCI-AML2 cells (OCI-AML2 + NPM1-mA) were generated. a Cytoplasmic localization of NPM1-mA in the OCI-AML2 stably expressing Flag-GFP tagged NPM1-mA. Cytospun cells were fixed and stained with DAPI. b The level of NPM1-mA in leukemia cells were measured by western blot analysis. [file 13046_2021_2122_MOESM6_ESM.pdf]
