# Supplementary material for: Mutant NPM1-regulated lncRNA HOTAIRM1 promotes leukemia cell autophagy and proliferation by targeting EGR1 and ULK3
Source: J Exp Clin Cancer Res. 2021 Oct 6;40:312. doi: 10.1186/s13046-021-02122-2 (PMC8493742; doi:10.1186/s13046-021-02122-2)

Additional file 7: Figure S2. The expression levels of HOTAIRM1 in the nucleus and cytoplasm of leukemia cells were measured by qRT-PCR

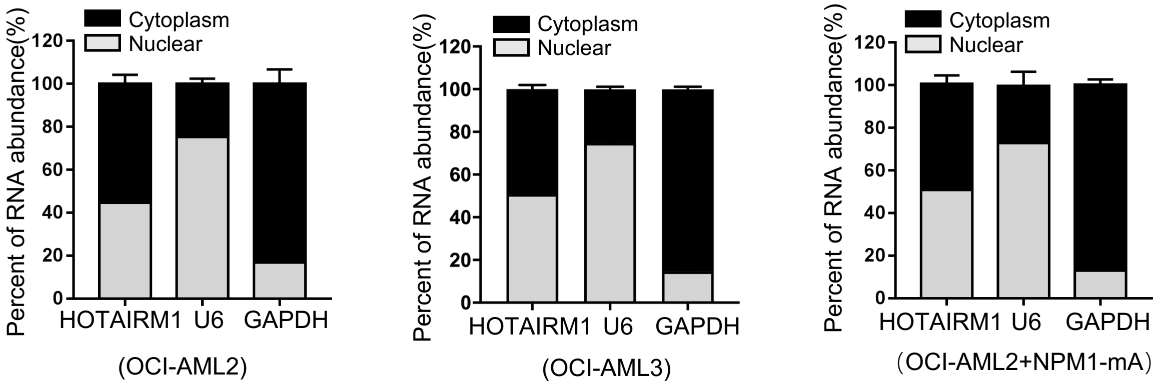

Supplement: Supplementary file 7 — Additional file 7 : Figure S2. The expression levels of HOTAIRM1 in the nucleus and cytoplasm of leukemia cells were measured by qRT-PCR. [file 13046_2021_2122_MOESM7_ESM.pdf]
