# Supplementary material for: Mutant NPM1-regulated lncRNA HOTAIRM1 promotes leukemia cell autophagy and proliferation by targeting EGR1 and ULK3
Source: J Exp Clin Cancer Res. 2021 Oct 6;40:312. doi: 10.1186/s13046-021-02122-2 (PMC8493742; doi:10.1186/s13046-021-02122-2)

Additional file 8: Figure S3. NPM1-mA expression in leukemia cells in the different treatment groups

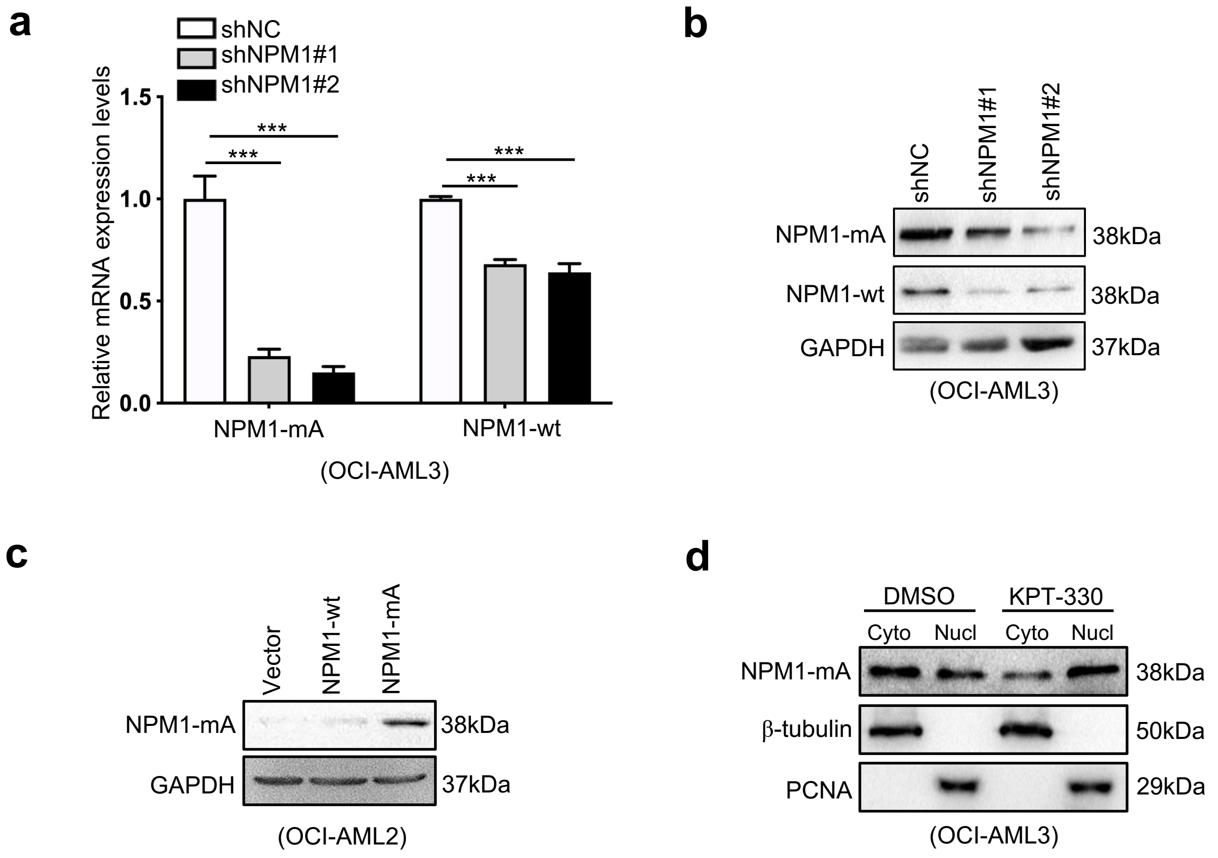

Supplement: Supplementary file 8 — Additional file 8 : Figure S3. NPM1-mA expression in leukemia cells in the different treatment groups. a The efficiency of RNA interference-mediated knockdown of NPM1-mA expression was determined by qRT-PCR. b The efficiency of RNA interference-mediated knockdown of NPM1-mA expression was determined by western blotting. c NPM1-mA protein levels in NPM1-mA enforced OCI-AML2 cells. d Western blot analysis of NPM1-mA in OCI-AML3 cells treated with KPT-330. β-tubulin as the cytoplasmic control and PCNA as the nuclear control. The data are presented as the mean ± SD of three independent experiments. [file 13046_2021_2122_MOESM8_ESM.pdf]
