# Supplementary material for: Mutant NPM1-regulated lncRNA HOTAIRM1 promotes leukemia cell autophagy and proliferation by targeting EGR1 and ULK3
Source: J Exp Clin Cancer Res. 2021 Oct 6;40:312. doi: 10.1186/s13046-021-02122-2 (PMC8493742; doi:10.1186/s13046-021-02122-2)

Additional file 9: Figure S4. ChIP-qPCR analysis of NPM1 occupancy on the HOTAIRM1 promoter in leukemia cells

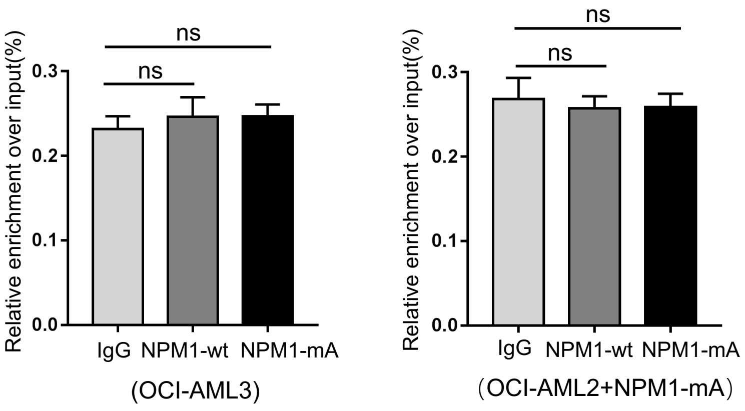

Supplement: Supplementary file 9 — Additional file 9 : Figure S4. ChIP-qPCR analysis of NPM1 occupancy on the HOTAIRM1 promoter in leukemia cells. [file 13046_2021_2122_MOESM9_ESM.pdf]
