# Supplementary material for: Mutant NPM1-regulated lncRNA HOTAIRM1 promotes leukemia cell autophagy and proliferation by targeting EGR1 and ULK3
Source: J Exp Clin Cancer Res. 2021 Oct 6;40:312. doi: 10.1186/s13046-021-02122-2 (PMC8493742; doi:10.1186/s13046-021-02122-2)

Additional file 10: Figure S5. Enrichment of KLF5 on the E2 fragment of the HOTAIRM1 promoter was measured by ChIP assays in leukemia cells

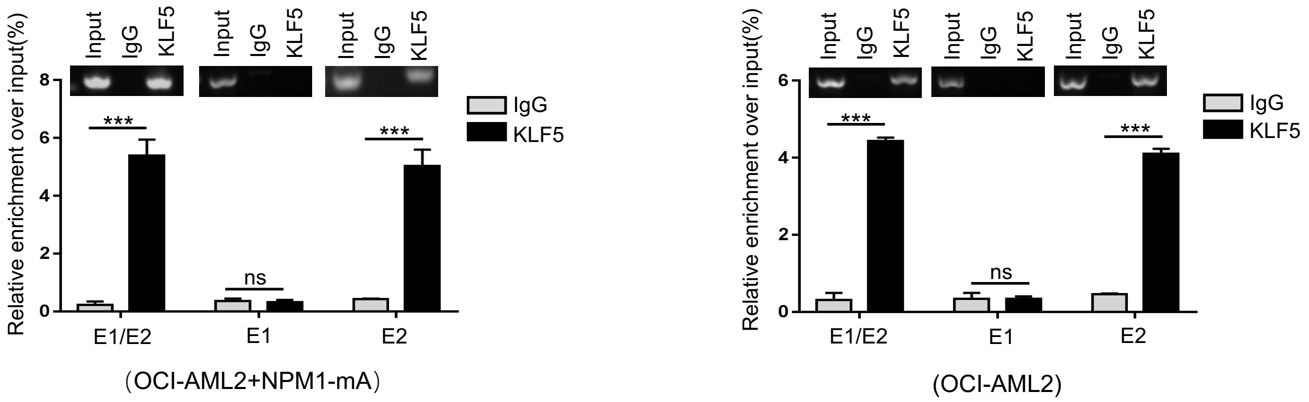

Supplement: Supplementary file 10 — Additional file 10 : Figure S5. Enrichment of KLF5 on the E2 fragment of the HOTAIRM1 promoter was measured by ChIP assays in leukemia cells. [file 13046_2021_2122_MOESM10_ESM.pdf]
