# Supplementary material for: Mutant NPM1-regulated lncRNA HOTAIRM1 promotes leukemia cell autophagy and proliferation by targeting EGR1 and ULK3
Source: J Exp Clin Cancer Res. 2021 Oct 6;40:312. doi: 10.1186/s13046-021-02122-2 (PMC8493742; doi:10.1186/s13046-021-02122-2)

Additional file 11: Figure S6. Co-IP assays were used to evaluate the binding of NPM1 and KLF5, NPM1-wt and WWP1 in OCI-AML3 cells

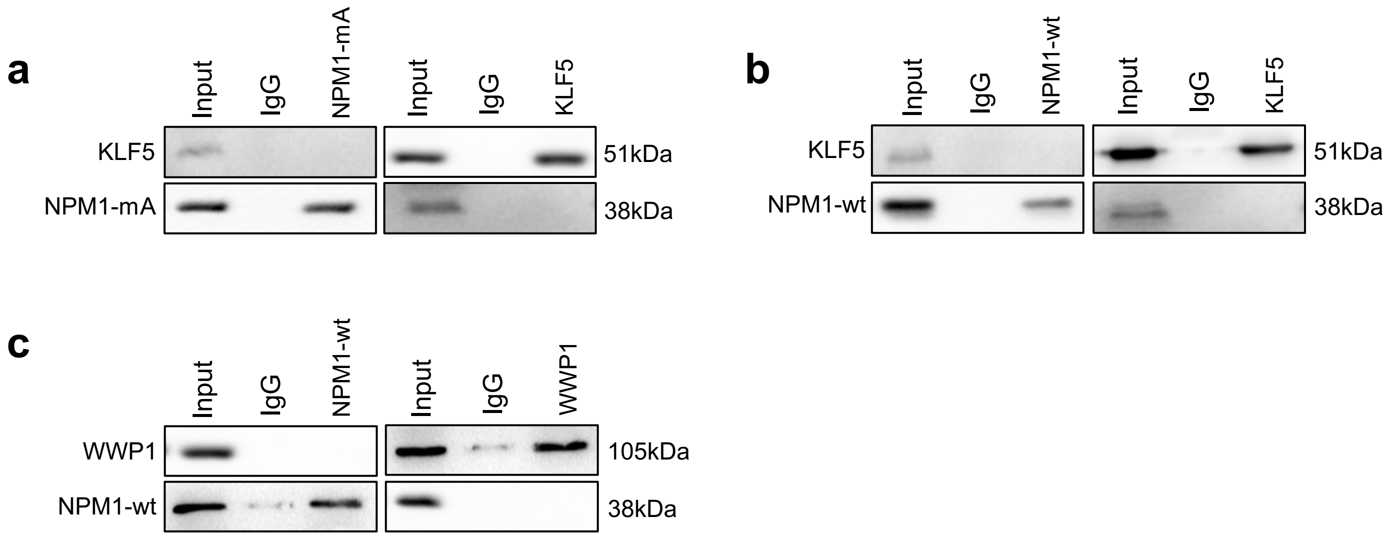

Supplement: Supplementary file 11 — Additional file 11 : Figure S6. Co-IP assays were used to evaluate the binding of NPM1 and KLF5, NPM1-wt and WWP1 in OCI-AML3 cells. a-b The interaction between NPM1-mA (a) or NPM1-wt (b) and KLF5 in OCI-AML3 cells were determined by IP assays. c IP assays were used to determine the binding of WWP1 to NPM1-wt in OCI-AML3 cells. [file 13046_2021_2122_MOESM11_ESM.pdf]
