# Supplementary figures and images for: Mutant NPM1-regulated lncRNA HOTAIRM1 promotes leukemia cell autophagy and proliferation by targeting EGR1 and ULK3
Source: J Exp Clin Cancer Res. 2021 Oct 6;40:312. doi: 10.1186/s13046-021-02122-2 (PMC8493742; doi:10.1186/s13046-021-02122-2)

**a**

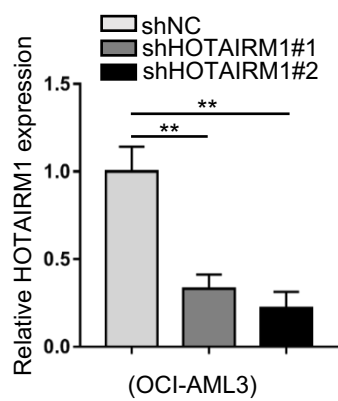

**b**

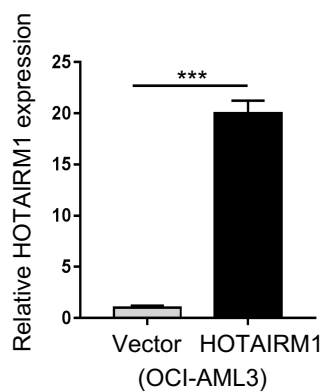

**c**

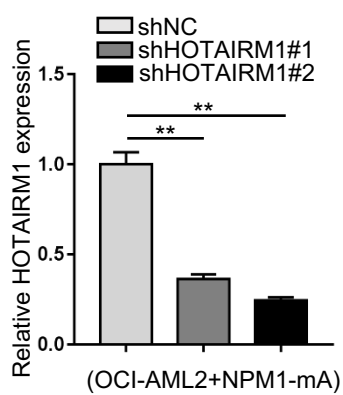

**d**

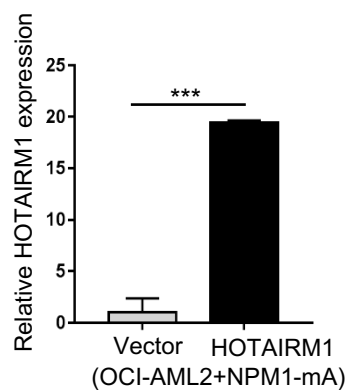

**e**

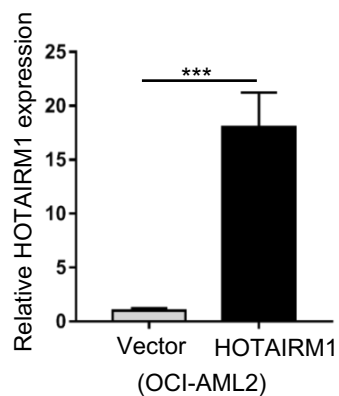

Supplement: Supplementary file 12 — Additional file 12 : Figure S7. HOTAIRM1 expression in the different transfected cells. a-b HOTAIRM1 expression in HOTAIRM1-silenced (a) and HOTAIRM1-enforced OCI-AML3 cells (b). c-d HOTAIRM1 expression in HOTAIRM1-silenced (c) as well as HOTAIRM1-enforced OCI-AML2 + NPM1-mA cells (d). e HOTAIRM1 expression in HOTAIRM1-enforced OCI-AML2 cells. [file 13046_2021_2122_MOESM12_ESM.pdf]

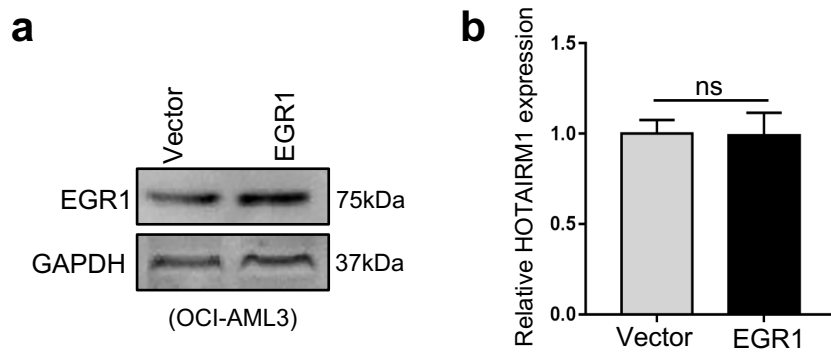

Supplement: Supplementary file 16 — Additional file 16 : Figure S11. EGR1 does not affect the expression of HOTAIRM1. a The efficiency of EGR1 overexpression in OCI-AML3 cells was validated by western blotting. b qRT-PCR was used to detect the expression of HOTAIRM1 in EGR1-enforced OCI-AML3 cells. The data are presented as the mean ± SD of three independent experiments. n.s. indicates no significant difference. [file 13046_2021_2122_MOESM16_ESM.pdf]
