# Supplementary material for: Mutant NPM1-regulated lncRNA HOTAIRM1 promotes leukemia cell autophagy and proliferation by targeting EGR1 and ULK3
Source: J Exp Clin Cancer Res. 2021 Oct 6;40:312. doi: 10.1186/s13046-021-02122-2 (PMC8493742; doi:10.1186/s13046-021-02122-2)

Additional file 13: Figure S8. HOTAIRM1 promotes autophagy and proliferation in transfected OCI-AML2+NPM1-mA leukemia cells

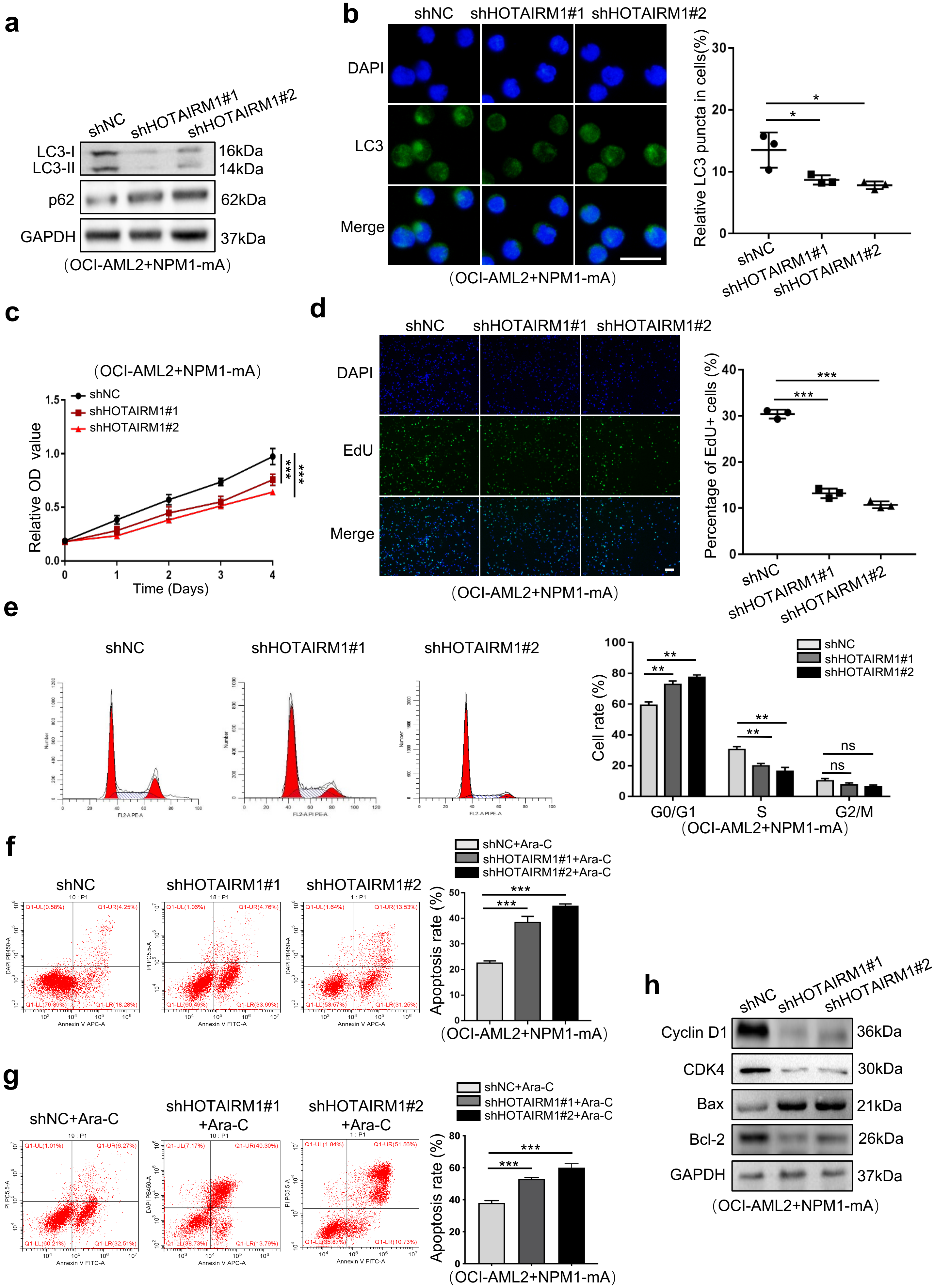

Additional file 13: Figure S8 Continued HOTAIRM1 promotes leukemia cell autophagy and proliferation in transfected OCI-AML2+NPM1-mA cells

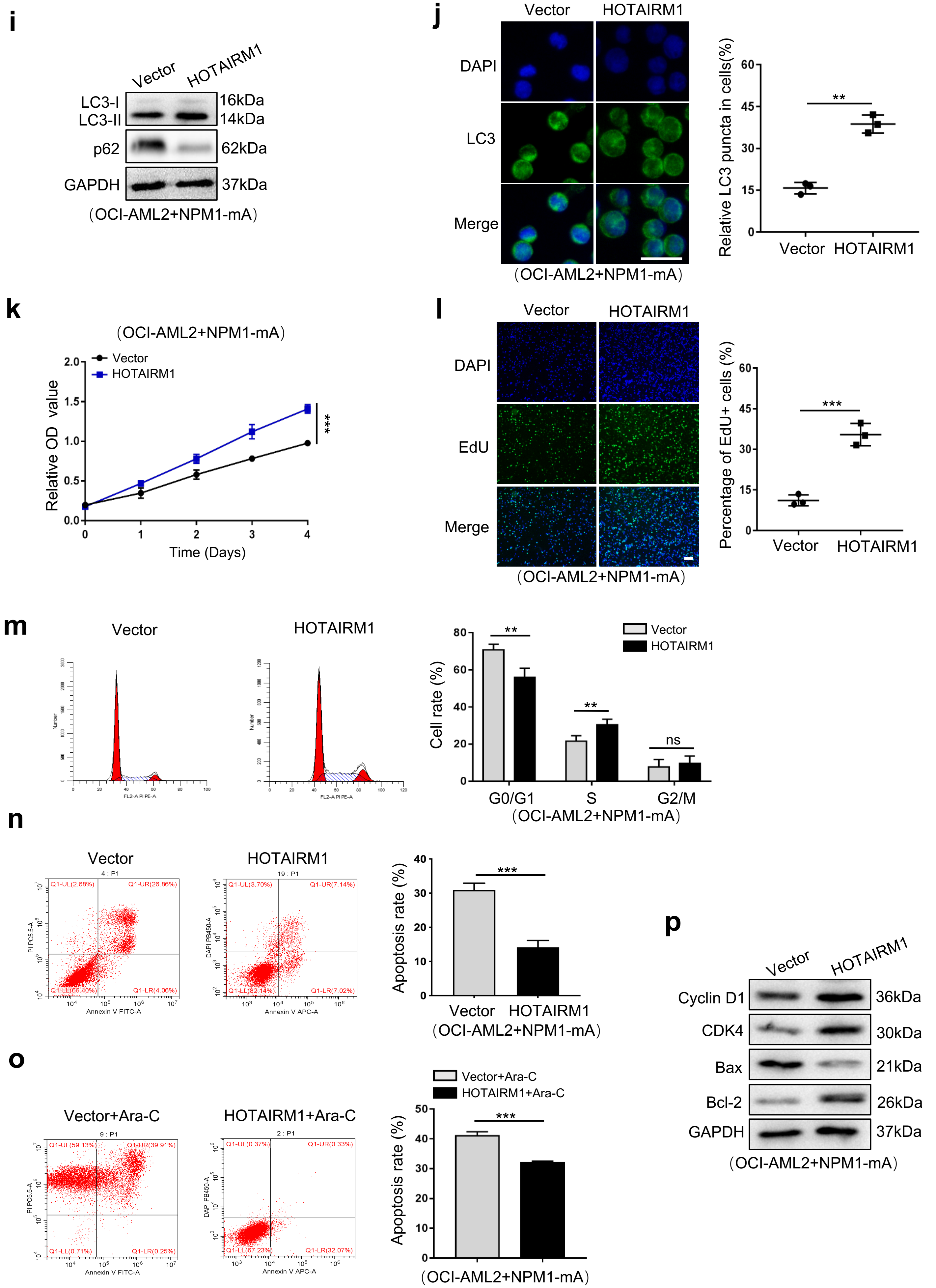

Supplement: Supplementary file 13 — Additional file 13 : Figure S8. HOTAIRM1 promotes autophagy and proliferation in transfected OCI-AML2 + NPM1-mA leukemia cells. a, i Western blot analysis of LC3-II and p62 levels in OCI-AML2 + NPM1-mA cells after transfection of shHOTAIRM1 (a) and HOTAIRM1 expression vector (i). b, j Representative microscopy images of LC3 puncta in HOTAIRM1-silenced OCI-AML2 + NPM1-mA (b) and HOTAIRM1-enforced OCI-AML2 + NPM1-mA (j). The bar graphs showed the quantification of the fluorescent puncta data. Scale bar: 25 μm. c, k Evaluation of cell viability in OCI-AML2 + NPM1-mA cells transfected with shHOTAIRM1 (c) or HOTAIRM1 plasmid (k) for indicated by CCK-8 assays. d, l Evaluation of cell proliferation in HOTAIRM1-silenced (d) and HOTAIRM1-enforced OCI-AML + NPM1-mA cells (l) for indicated by EdU assays. The bar graphs showed the percentage of EdU positive cells. Scale bar: 100 μm. e, m Flow cytometry was performed to assess cell cycle of HOTAIRM1-silenced (e) and HOTAIRM1-enforced OCI-AML2 + NPM1-mA cells (m). The bar graph shows the percentages of G0/G1-, S-, and G2/M-phase cells. f, n Flow cytometry was used to detect apoptosis of HOTAIRM1-silenced (f) and HOTAIRM1-enforced OCI-AML2 + NPM1-mA cells (n). LL, dead cells; UL, viable cells; LR, early apoptotic cells; UR, late apoptotic cells. g, o Flow cytometric analysis was performed to analyze apoptosis of OCI-AML2 + NPM1-mA cells after transfection with shHOTAIRM1 (g) or HOTAIRM1 plasmid (o), followed by treatment with 200 nM Ara-C for 48 h. h, p The protein levels of Cyclin D1, CDK4, Bax and Bcl-2 in HOweTAIRM1-silenced (h) and HOTAIRM1-enforced OCI-AML2 + NPM1-mA cells (p). The data are presented as the mean ± SD of three independent experiments. **P < 0.01, ***P < 0.001. n.s. indicates no significant difference. [file 13046_2021_2122_MOESM13_ESM.pdf]
