# Supplementary material for: Mutant NPM1-regulated lncRNA HOTAIRM1 promotes leukemia cell autophagy and proliferation by targeting EGR1 and ULK3
Source: J Exp Clin Cancer Res. 2021 Oct 6;40:312. doi: 10.1186/s13046-021-02122-2 (PMC8493742; doi:10.1186/s13046-021-02122-2)

Additional file 14: Figure S9. HOTAIRM1 promotes autophagy and proliferation in transfected OCI-AML2 leukemia cells

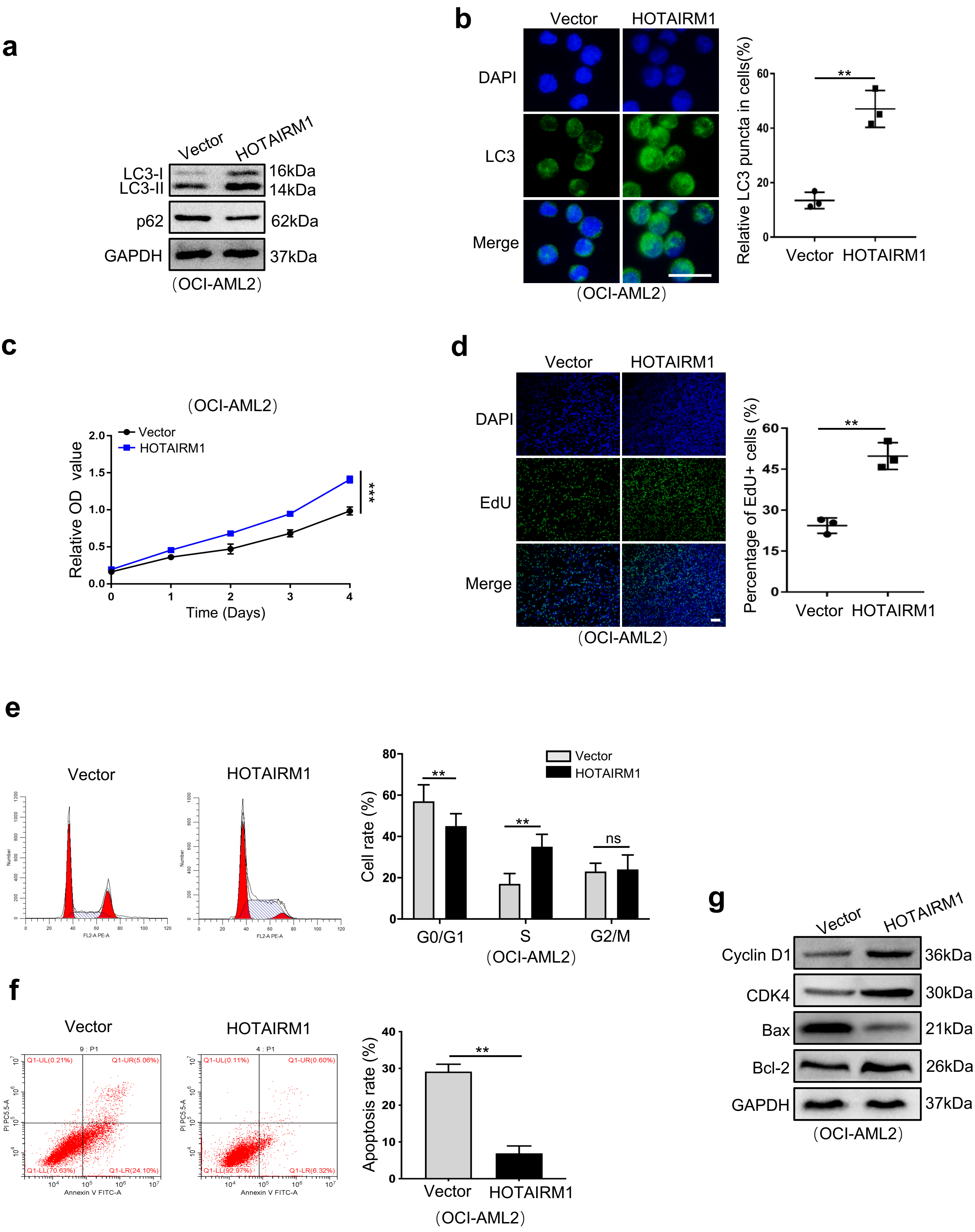

Supplement: Supplementary file 14 — Additional file 14 : Figure S9. HOTAIRM1 promotes autophagy and proliferation in transfected OCI-AML2 leukemia cells. a Western blot analysis of LC3-II and p62 levels in OCI-AML2 cells after transfection of HOTAIRM1 expression vector. b Representative microscopy images of LC3 puncta in HOTAIRM1-enforced OCI-AML2 cells. The bar graphs showed the quantification of the fluorescent puncta data. Scale bar: 25 μm. c Evaluation of cell viability in OCI-AML2 cells transfected with HOTAIRM1 plasmid for indicated by CCK-8 assays. d Evaluation of cell proliferation in HOTAIRM1-enforced OCI-AML2 cells for indicated by EdU assays. The bar graphs showed the percentage of EdU positive cells. Scale bar: 100 μm. e Flow cytometry was performed to assess cell cycle of HOTAIRM1-enforced OCI-AML2 cells. The bar graph shows the percentages of G0/G1-, S-, and G2/M-phase cells. f Flow cytometry was used to detect apoptosis of HOTAIRM1-enforced OCI-AML2 cells. LL, dead cells; UL, viable cells; LR, early apoptotic cells; UR, late apoptotic cells. g The protein levels of Cyclin D1, CDK4, Bax and Bcl-2 in HOTAIRM1-enforced OCI-AML2 cells. The data are presented as the mean ± SD of three independent experiments. **P < 0.01, ***P < 0.001. n.s. indicates no significant difference. [file 13046_2021_2122_MOESM14_ESM.pdf]
