# Supplementary material for: Mutant NPM1-regulated lncRNA HOTAIRM1 promotes leukemia cell autophagy and proliferation by targeting EGR1 and ULK3
Source: J Exp Clin Cancer Res. 2021 Oct 6;40:312. doi: 10.1186/s13046-021-02122-2 (PMC8493742; doi:10.1186/s13046-021-02122-2)

Additional file 15: Figure S10. HOTAIRM1 expression in HOTAIRM1-silenced OCI-AML3 cells transfected with different vectors

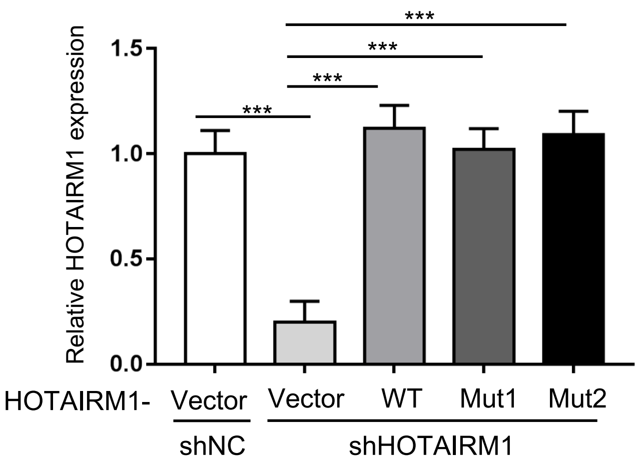

Supplement: Supplementary file 15 — Additional file 15 : Figure S10. HOTAIRM1 expression in HOTAIRM1-silenced OCI-AML3 cells transfected with different vectors. [file 13046_2021_2122_MOESM15_ESM.pdf]
