# Supplementary material for: Mutant NPM1-regulated lncRNA HOTAIRM1 promotes leukemia cell autophagy and proliferation by targeting EGR1 and ULK3
Source: J Exp Clin Cancer Res. 2021 Oct 6;40:312. doi: 10.1186/s13046-021-02122-2 (PMC8493742; doi:10.1186/s13046-021-02122-2)

**Additional file 17: Figure S12.** Screening for the ubiquitin ligase of EGR1

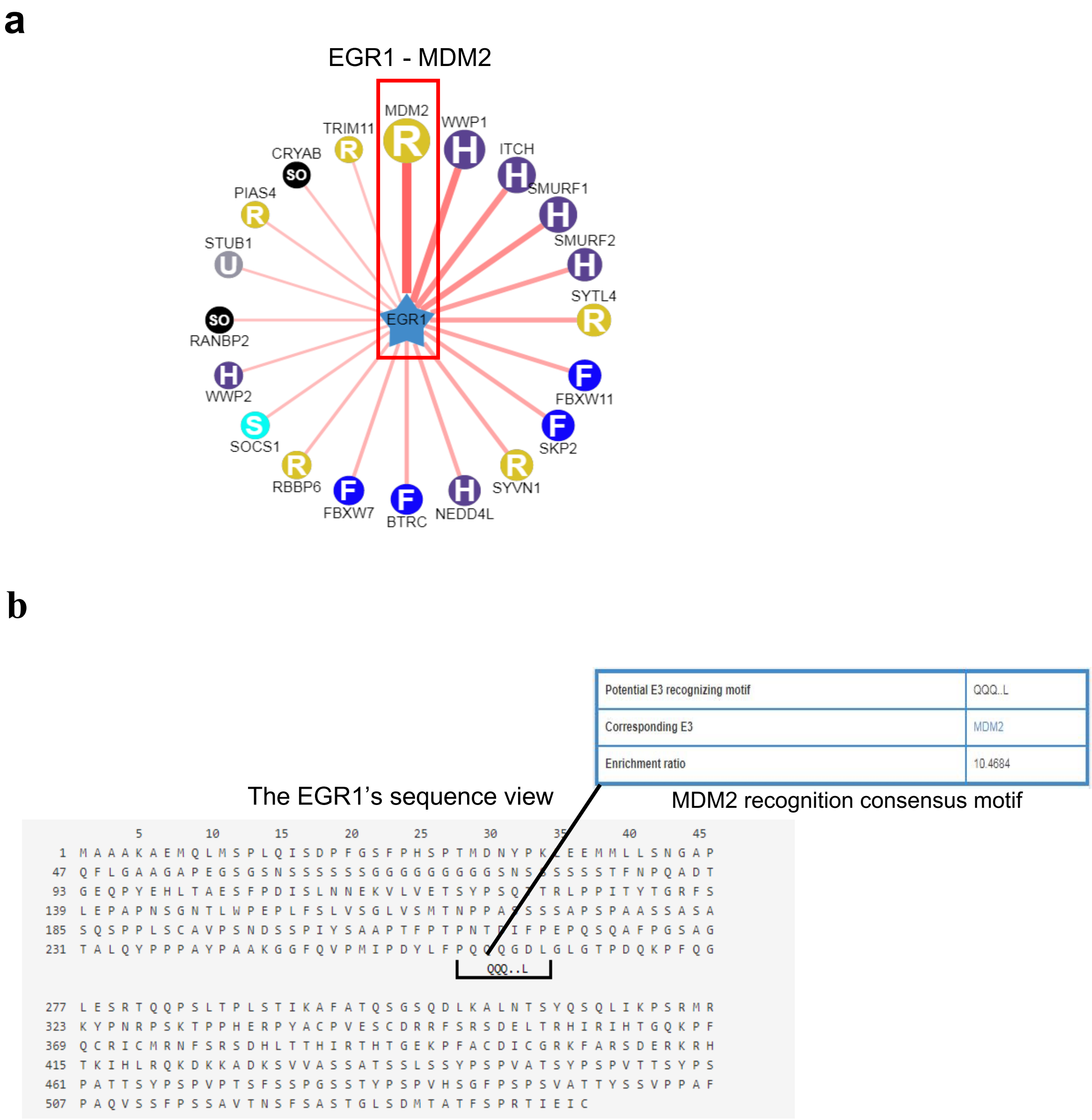

Supplement: Supplementary file 17 — Additional file 17 : Figure S12. Screening for the ubiquitin ligase of EGR1. a Network view of the predicted E3 ubiquitin ligase for EGR1. b Recognition motif for the potential E3 ubiquitin ligase MDM2 in the EGR1 sequence. [file 13046_2021_2122_MOESM17_ESM.pdf]
