# Supplementary material for: Mutant NPM1-regulated lncRNA HOTAIRM1 promotes leukemia cell autophagy and proliferation by targeting EGR1 and ULK3
Source: J Exp Clin Cancer Res. 2021 Oct 6;40:312. doi: 10.1186/s13046-021-02122-2 (PMC8493742; doi:10.1186/s13046-021-02122-2)

Additional file 18: Figure S13. Nuclear HOTAIRM1 promotes cell cycle progression and inhibits apoptosis in leukemia cells through EGR1

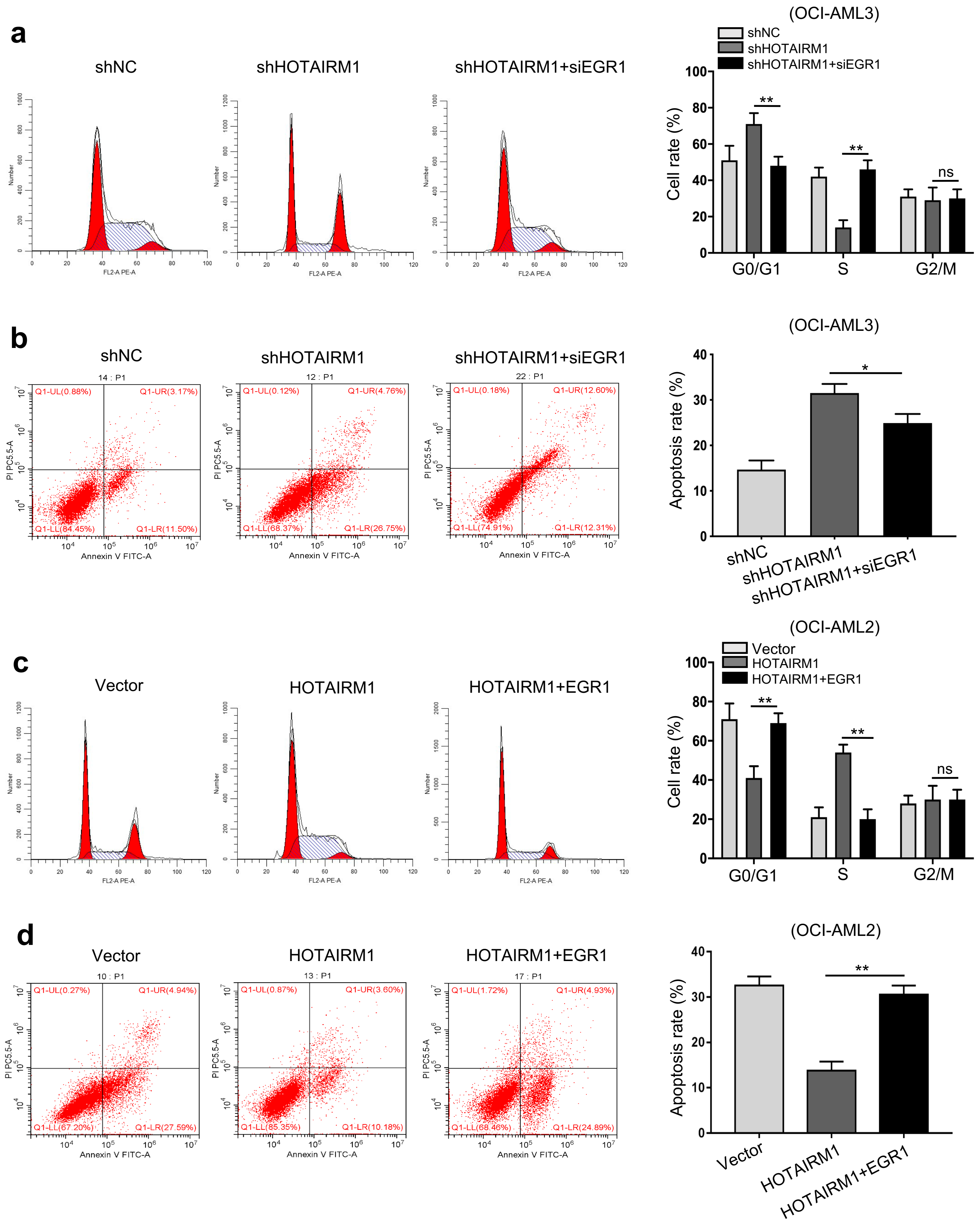

Supplement: Supplementary file 18 — Additional file 18 : Figure S13. Nuclear HOTAIRM1 promotes cell cycle progression and inhibits apoptosis in leukemia cells through EGR1. a, c Flow cytometry was used to determine and compare the differences in cell cycle progression in differently transfected groups of OCI-AML3 (a) and OCI-AML2 cells (c). b, d Flow cytometry was used to determine and compare differences in apoptosis in differently transfected groups of OCI-AML3 (b) and OCI-AML2 cells (d). The data are presented as the mean ± SD of three independent experiments. *P < 0.05, **P < 0.01. n.s. indicates no significant difference. [file 13046_2021_2122_MOESM18_ESM.pdf]
