# Supplementary material for: Mutant NPM1-regulated lncRNA HOTAIRM1 promotes leukemia cell autophagy and proliferation by targeting EGR1 and ULK3
Source: J Exp Clin Cancer Res. 2021 Oct 6;40:312. doi: 10.1186/s13046-021-02122-2 (PMC8493742; doi:10.1186/s13046-021-02122-2)

Additional file 19: Figure S14. Cytoplasmic HOTAIRM1 promotes leukemia cell autophagy and proliferation through ULK3

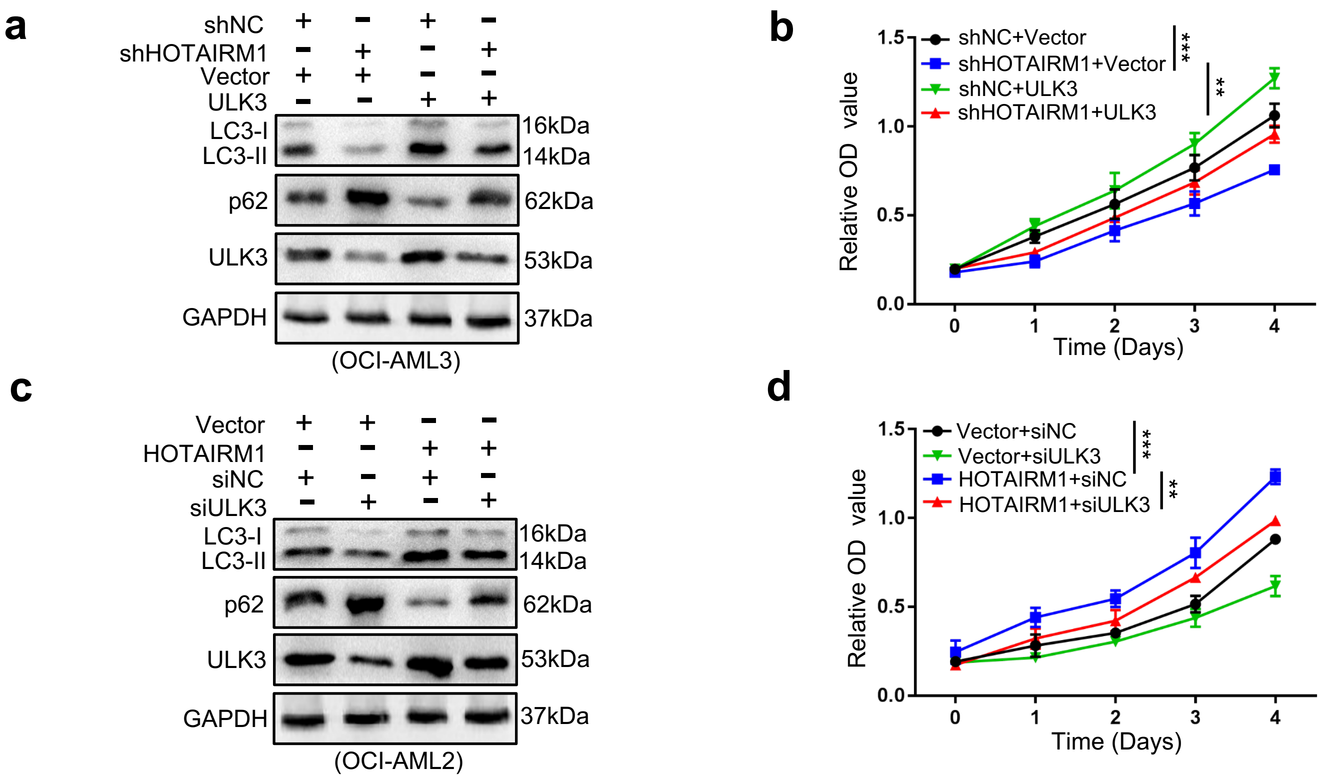

Supplement: Supplementary file 19 — Additional file 19 : Figure S14. Cytoplasmic HOTAIRM1 promotes leukemia cell autophagy and proliferation through ULK3. a, c Western blot analysis of LC3-II, p62 and ULK3 levels in HOTAIRM1-silenced OCI-AML3 cells following ULK3 overexpression (a) and HOTAIRM1-overexpressed OCI-AML2 cells following ULK3 knockdown (c). b, d CCK-8 assays were used to analyze cell proliferation in transfected OCI-AML3 (b) and OCI-AML2 cells (d). The data are presented as the mean ± SD of three independent experiments. *P < 0.05, **P < 0.01, ***P < 0.001. n.s. indicates no significant difference. [file 13046_2021_2122_MOESM19_ESM.pdf]
