# Supplementary material for: Mutant NPM1-regulated lncRNA HOTAIRM1 promotes leukemia cell autophagy and proliferation by targeting EGR1 and ULK3
Source: J Exp Clin Cancer Res. 2021 Oct 6;40:312. doi: 10.1186/s13046-021-02122-2 (PMC8493742; doi:10.1186/s13046-021-02122-2)

Additional file 20: Figure S15. Cytoplasmic HOTAIRM1 promotes cell cycle progression and inhibits apoptosis in leukemia cells through ULK3

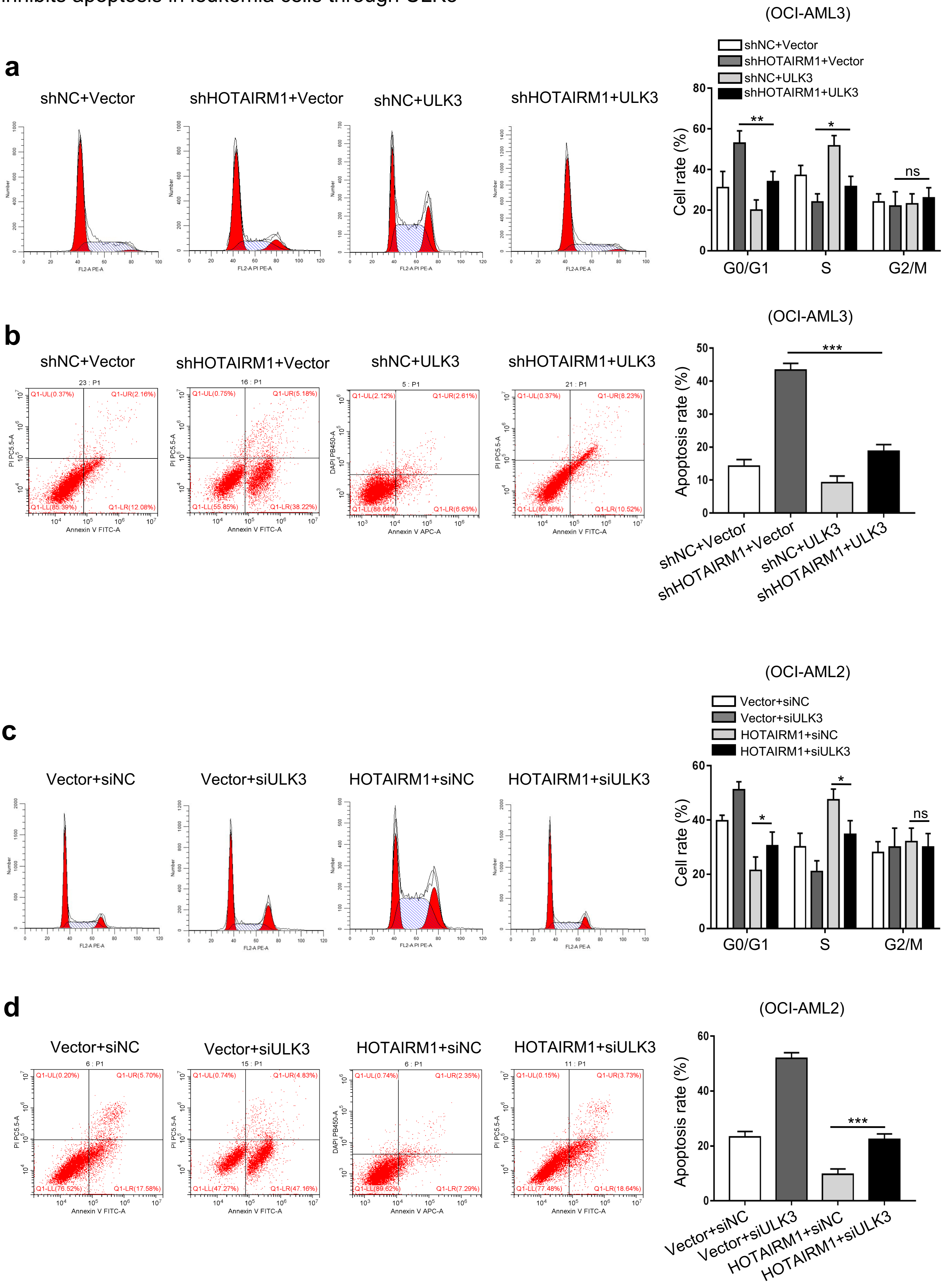

Supplement: Supplementary file 20 — Additional file 20 : Figure S15. Cytoplasmic HOTAIRM1 promotes cell cycle progression and inhibits apoptosis in leukemia cells through ULK3. a-b Flow cytometry was used to analyze the changes in cell the cycle (a) and apoptosis (b) in HOTAIRM1-silenced OCI-AML3 cells following ULK3 upregulation. c-d Flow cytometry was used to analyze the change in the cell cycle (c) and apoptosis (d) in HOTAIRM1-overexpressed OCI-AML2 cells following ULK3 downregulation. The data are presented as the mean ± SD of three independent experiments. *P < 0.05, **P < 0.01, ***P < 0.001. n.s. indicates no significant difference. [file 13046_2021_2122_MOESM20_ESM.pdf]
